# Pan-viral ORFs discovery using Massively Parallel Ribosome Profiling

**DOI:** 10.1126/science.ado6670

**Published:** 2025-06-12

**Authors:** Shira Weingarten-Gabbay, Matthew R. Bauer, Alexandra C. Stanton, Yingpu Yu, Catherine A. Freije, Nicole L. Welch, Chloe K. Boehm, Susan Klaeger, Eva K. Verzani, Daniel López, Lisa E. Hensley, Karl R. Clauser, Steven A. Carr, Jennifer G. Abelin, Charles M. Rice, Pardis C. Sabeti

**Affiliations:** 1 Broad Institute of MIT and Harvard, Cambridge, MA, USA; 2 Department of Organismic and Evolutionary Biology, Harvard University, Cambridge, MA, USA; 3 Laboratory of Virology and Infectious Disease, The Rockefeller University, New York, NY, USA; 4 Harvard Program in Biological and Biomedical Sciences, Division of Medical Sciences, Harvard Medical School, Boston, Massachusetts, USA; 5 Harvard Program in Virology, Harvard Medical School, Boston, MA, USA; 6 Howard Hughes Medical Institute, Chevy Chase, MD, USA; 7 Unidad de Presentación y Regulación Inmunes, Centro Nacional de Microbiología, Instituto de Salud Carlos III, Majadahonda (Madrid), Spain; 8 Zoonotic and Emerging Disease Research Unit, National Bio and Agro-Defense Facility, U.S. Department of Agriculture, Agricultural Research Service (ARS), Manhattan, KS, USA; 9 Massachusetts Consortium on Pathogen Readiness, Boston, MA, USA; 10 Department of Immunology and Infectious Disease, Harvard T.H. Chan School of Public Health, Boston, MA, USA; 11 Current affiliation: Department of Microbiology, Harvard Medical School, Boston, MA, USA; 12 Current affiliation: Harvard Law School, Harvard University, Cambridge, MA, USA

## Abstract

Defining viral proteomes is crucial to understanding viral life cycles and immune recognition; yet the landscape of translated regions remains unknown for most viruses. We have developed Massively Parallel Ribosome Profiling (MPRP) to determine open reading frames (ORFs) across tens of thousands of designed oligonucleotides. MPRP identified 4,208 unannotated ORFs in 679 human-associated viral genomes. We found viral peptides originating from detected non-canonical ORFs presented on class I human leukocyte antigen (HLA-I) in infected cells, and hundreds of upstream ORFs (uORFs) that likely modulate translation initiation of viral proteins. The discovery of viral ORFs across a wide range of viral families, including highly pathogenic viruses, expands the repertoire of vaccine targets, and reveals potential *cis*-regulatory sequences.

## INTRODUCTION

Despite advances in sequencing viral genomes, functional annotations of these genomes have lagged behind. Beyond the annotated canonical open reading frames (ORFs), viral genomes encode non-canonical ORFs that do not fulfill the classical definition of ORFs, i.e., they do not start with an ATG and/or are shorter than 100 amino acids (aa). These non-canonical ORFs and the resulting microproteins modulate viral infection (1–3), contribute to the immune response to viruses (4–8), and regulate gene expression (9–11). However, the deviation from classical ORF features complicates their detection by computational approaches, and relies mostly on experimental measurements.

The development of ribosome profiling (also termed Ribo-seq) has transformed our ability to detect translated regions across genomes (12). Ribosome profiling utilizes deep sequencing of ribosome-bound mRNA fragments to determine ribosome occupancy, indicating translated regions at single nucleotide resolution. It has uncovered many non-canonical ORFs in mammalian cells, yeast, bacteria, and viruses including upstream ORFs (uORFs) and upstream-overlapping ORFs (uoORFs) in 5’UTRs, short ORFs in non-coding RNAs, and overlapping internal ORFs in annotated coding sequence (iORFs) (13, 14).

The landscape of translated regions, however, is still unknown for the majority of viruses. Ribosome profiling has only been used to profile a handful of viruses due to the following challenges: each virus demands a unique culturing system; some viruses cannot be cultured in the lab; and highly pathogenic viruses require high-containment facilities. Moreover, most viruses are genetically diverse, necessitating a method that can evaluate multiple variants in parallel.

We have leveraged an oligo synthesis library (15–17) and combined it with ribosome profiling to perform pan-viral ORF discovery. We measured the translation of 20,170 synthetic sequences from 679 viral genomes in two human cell lines, under stress conditions associated with viral infection, and when expressed by cap-dependent or IRES-dependent translation. We estimated ORF discovery using the annotated coding sequences (CDSs) and previously reported non-canonical ORFs. We compared ribosome footprints in the synthetic library and natural infection with four viruses. We then examined the function of the detected ORFs in two processes: HLA-I antigen presentation and uORF-mediated translation regulation.

## RESULTS

### Massively Parallel Ribosome Profiling to identify ORFs

**Figure 1.**
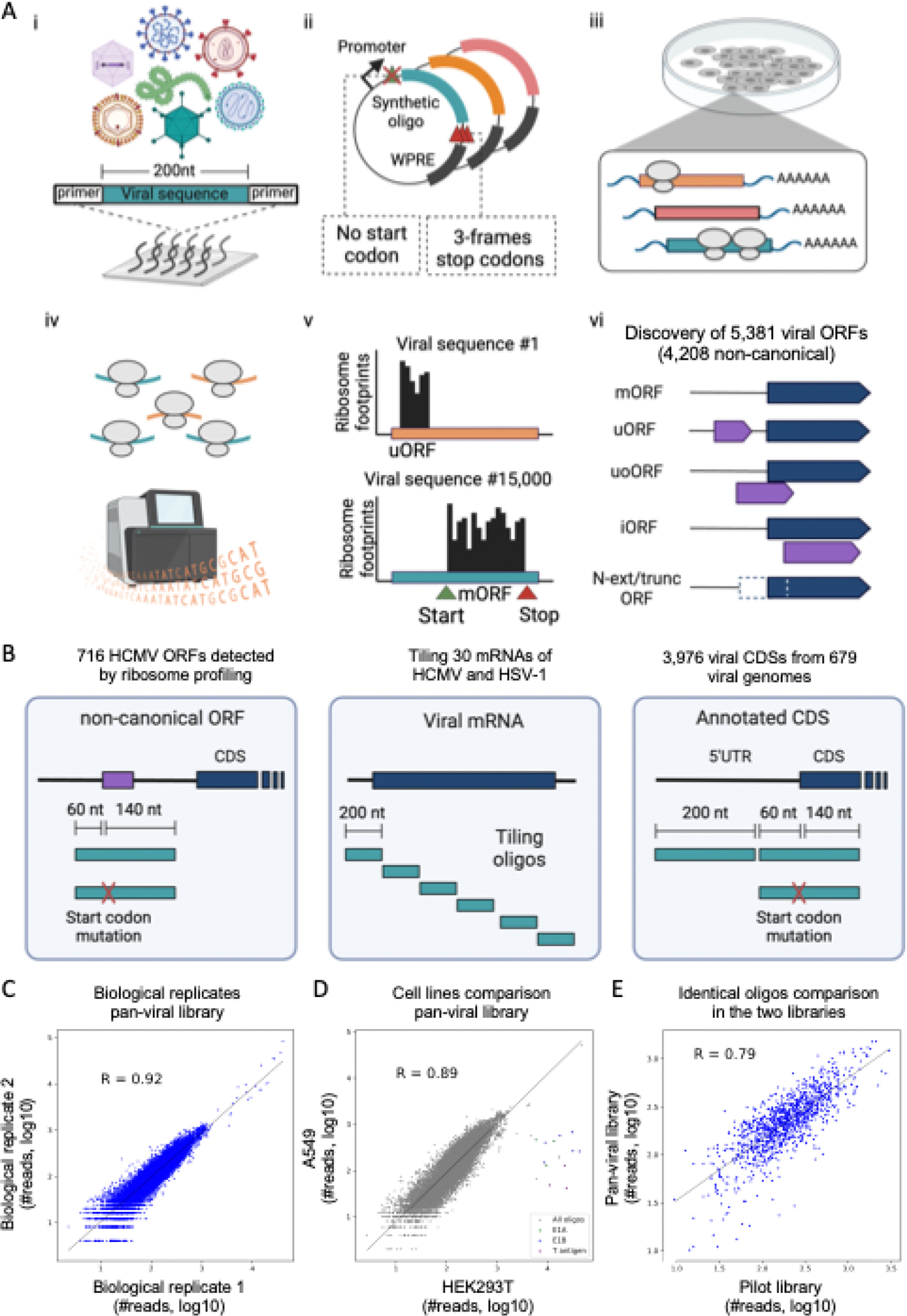
Design of oligonucleotide synthetic library and MPRP measurements **(A)** Illustration of the Massively Parallel Ribosome Profiling experiment (MPRP). (i) Synthetic library amplification using constant primers. (ii) Cloning library into overexpression vector. (iii) Transient transfection of plasmid pool into HEK293T or A549 cells for 24 h. (iv) Treating cells with either LTM or CHX and performing ribosome profiling protocol. (v) Mapping deep sequencing reads to the synthetic library. (vi) Inferring translated ORFs using PRICE (19). **(B)** Design of the tested synthetic oligonucleotides: (i) ORFs identified by ribosome profiling in infected cells with either intact start codon or a GCC mutation. (ii) Tiling oligos encompassing complete viral transcripts. (iii) Oligos spanning the 5’UTR and the first 140 nt of annotated viral CDSs. For the region containing the CDS, two oligos were designed: the wild-type sequence and a start codon mutated oligo. **(C)** Comparing the number of ribosome footprints mapped to 15,000 oligos in two biological replicates in HEK293T cells. R=0.92, Pearson correlation. **(D)** Comparing the number of ribosome footprints mapped to 15,000 oligos in HEK293T and A549 cells. R=0.89, Pearson correlation. Showing in color oligos with shared sequences with the adenoviral E1A/B genes and the SV40 large T-antigen endogenously expressed by HEK293T cells. **(E)** Comparing the number of ribosome footprints mapped to 1,163 identical oligos in two synthetic libraries in HEK293T cells. R=0.79, Pearson correlation.

We developed Massively Parallel Ribosome Profiling (MPRP) to measure translated regions across hundreds of human viruses (Figure 1A). We used oligonucleotide library synthesis technology to encapsulate thousands of viral sequences in a single pooled experiment. Each oligo contained 200nt of viral sequence, flanked by constant primers. We cloned the library into an overexpression plasmid downstream of a CMV promoter and upstream of a Woodchuck Hepatitis Virus Posttranscriptional Regulatory Element (WPRE) used to further enhance expression. To monitor the translation of ORFs in the designed oligos, we excluded ATG start codons on the plasmid. We transfected the pooled library plasmid into HEK293T or A549 cells. We then performed a modified protocol of ribosome profiling (18) after treating cells with either cycloheximide (CHX) to inhibit elongating ribosomes, or lactimidomycin (LTM) to inhibit initiating ribosomes. We mapped deep sequencing reads representing ribosome footprints to the synthetic library and identified ORFs using PRICE, a computational method for the detection of ORFs in ribosome profiling experiments (19).

We tested the quality of ribosome footprints by examining host-mapped reads, showing the expected read length distribution, footprint enrichment in the CDS, and tri-nucleotide periodicity (Figure S1). To verify that our experimental system captures the translation of an ORF embedded within a synthetic oligo, we performed ribosome profiling on cells transiently transfected with a full-length or truncated Green Fluorescent Protein (GFP) (Figure S2).

We designed a ‘pilot’ library to estimate viral ORF discovery using MPRP. We tiled 30 mRNAs annotated in the genomic datasets of HCMV and HSV-1 (Figure S3A). We observed the expected low occupancy in the 5’UTR and enrichment of ribosomes at the start codon (Figure S3B). However, we also observed high occupancy in oligos along the CDS that do not contain the CDS start codon. In the context of the full transcript, these alternative initiation sites are preceded by multiple start codons, making it unlikely that the ribosome will initiate at these positions. We reasoned that a tiling approach may result in false-positive ORF discovery and that MPRP has higher accuracy at the beginning of annotated CDSs and 5’UTRs. Thus, we decided to focus on this region for the design of the pan-viral library, which encompasses the majority of ORF initiation sites (13).

We designed a ‘pan-viral’ library of 15,000 oligos to screen for novel ORFs in the 5’UTRs and the beginning of the CDS of 3,976 genes in 679 viral genomes. For each gene, we designed three oligos: a wild type oligo containing 60 nt of the 5’UTR and 140nt of the CDS, a mutated oligo in which the annotated start codon was mutated to GCC, and a farther upstream oligo in the 5’UTR region, starting −260 nt relative to the CDS start codon (Figure 1B).

To gauge the reproducibility of the MPRP measurements, we compared ribosome occupancy on the synthetic oligos in different experiments. Measurements of the pilot and the pan-viral libraries were consistent between biological replicates in HEK293T cells (R=0.81 and R=0.92, respectively, Figure S4 and 1C). We found high agreement between ribosome occupancies on the pan-viral library in HEK293T and A549 cells (R=0.89, Figure 1D). We also designed a set of 1,033 oligos with identical sequences in both the pilot and pan-viral libraries and found good agreement between oligos that were independently synthesized, cloned, and measured (R=0.79, Figure 1E).

To imitate the cellular environment in infected cells, we repeated MPRP when inducing stress. We treated cells with poly(I:C) to model viruses that form double-strand RNA, and Thapsigargin to induce endoplasmic reticulum (ER) stress. We confirmed the expected phosphorylation of translation regulators (PKR and eIF2alpha, Figure S5A) and innate immune sensors (IRF3 and STAT1, Figure S5B). Interestingly, we observed phosphorylation of PKR, eIF2alpha, IRF3, and STAT1 in cells expressing a plasmid without treatment, suggesting that transient transfection by itself induces a similar stress response. Accordingly, we found a significant agreement between MPRP measurements in non-treated and poly(I:C)-treated A549 and HEK293T cells (R=0.773 and R=0.767, respectively, Figures S5C, 5D). These results indicate that sequences tested in MPRP are exposed to a cellular environment associated with viral infection.

MPRP measurements on the pan-viral library uncovered 5,381 ORFs including 4,208 non-canonical ORFs (Table S1) for further study.

### Estimating ORF discovery using annotated viral coding sequences

**Figure 2.**
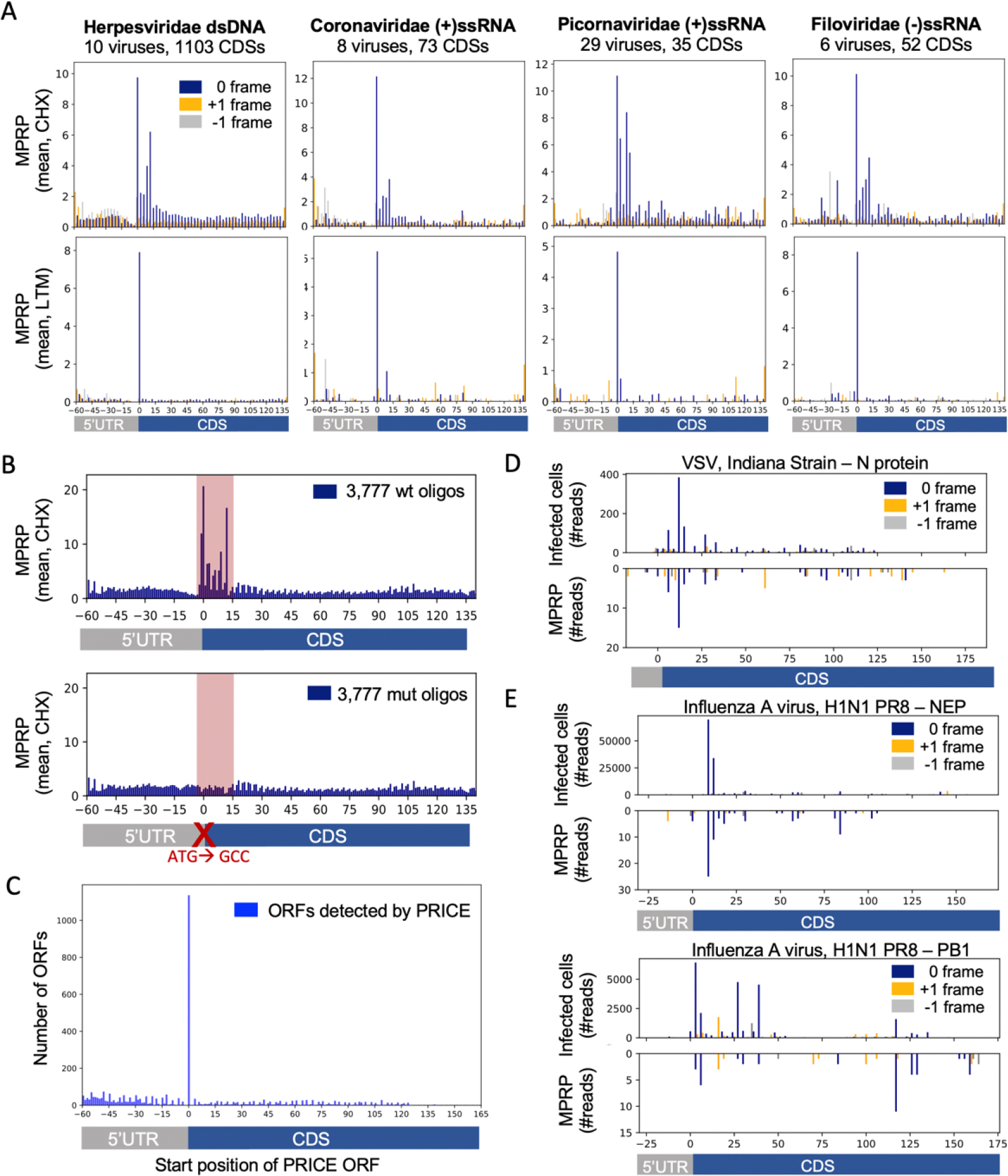
Annotated CDSs measurements in MPRP and infected cells **(A)** Metagene analysis for four viral families with CHX and LTM inhibitors showing the average ribosome footprints in each position. See Figures S6 and S7 for 21 viral families. **(B)** Comparing the average number of ribosome footprints between oligos containing the wt start codon (upper graph) to those in which the annotated start codon was mutated to GCC (lower graph). Shown are the average ribosome footprints in each position across 3,777 oligos. **(C)** ORF discovery using PRICE. Showing the number of ORFs detected in each position (ORF start position). **(D)** Mirror plot showing the number of ribosome footprints mapped to the first 200nt of the Nucleocapsid transcript in VSV-infected cells and MPRP. **(E)** Similar to (D) for IAV NEP and PB1 transcripts.

To estimate the detection of ORFs, we examined the distribution of ribosome footprints across oligos representing the first 140nt of annotated viral CDSs (Figure 1B). We performed metagene analysis for all the CDSs from each family by computing the average ribosome footprints in each position relative to the annotated start codon. In the majority of the 21 viral families tested, we found the expected enrichment of ribosome footprints in the CDS and in the correct reading frame, indicating a robust identification of annotated CDSs in MPRP across different viruses (Figures 2A, S6).

We next examined ribosome footprints in the presence of an LTM inhibitor, which inhibits initiating 80S complexes. We found a clear enrichment in the annotated start codons for 19 of the 21 viral families tested (Figures 2A, S7). Using reverse genetics, we found a significant 3.7-fold reduction in the number of footprints on 3,777 oligos in which we mutated the annotated start codon to GCC (p<10^−288^, Figures 2B, S8).

We used the annotated CDSs to assess the performance of our computational pipeline for inferring ORFs in the MPRP experiment. When running PRICE, we did not indicate which viral oligos contain annotated CDSs in order to evaluate their discovery in an unbiased fashion. PRICE successfully captured 1,136 of 3,976 viral CDSs initiating at the annotated start codon (28%, accounting for 31% of the total ORFs detected by PRICE, Figure 2C), with a high correlation between the number of reads in two biological replicates (R=0.93, Figure S9). The observance of 28% of the annotated CDSs may represent inherent limitations of the MPRP, assaying the translation of viral sequences outside the context of viral infection (see Discussions). Nevertheless, we observe specific enrichment at the annotated start codon (15.3-fold higher than the next most abundant position).

Since many viruses rely on Internal Ribosome Entry Sites (IRESs) for protein translation, we tested whether the mode of ribosome recruitment affects ORF discovery. We cloned the pan-viral library into a bicistronic plasmid downstream of the EMCV or the Polio IRES and repeated MPRP in HEK293T cells (Figure S10A, S11, Tables S2, S3). We observed the expected enrichment of ribosomes on annotated CDSs and a reduction in start codon mutated oligos when expressing the library from both IRESs (Figure S10B). We found a significant overlap between PRICE-detected CDSs in the cap-dependent and IRES-dependent MPRPs (hypergeometric p-value<10^−20^ and p-value<10^−8^ for EMCV and Polio IRES, respectively) and agreement between the number of ribosome footprints on shared ORFs (R=0.75 and R=0.83 for EMCV and Polio IRES, respectively, Figure S10C, S10D). Finally, we found a similar pattern of ribosome footprints in the cap- and IRES-dependent MPRPs on CDSs of viruses that rely on IRESs for translation initiation from the Flaviviridae and Picornaviridae families (Figure S10E, S10F). These results suggest that at least for some proteins, ORF discovery was independent of the mechanism by which the ribosome was recruited to the transcript.

We then compared the pattern of ribosome footprints in MPRP and native viral infection. We performed ribosome profiling in cells infected with vesicular stomatitis virus (VSV, Indiana strain), influenza A virus (IAV, Puerto Rico 8 H1N1 strain), and Hepatitis C Virus (HCV, genotype 2) (Figure S12). We found a high congruence between the location of footprints originating from MPRP and infected cells in CDSs from VSV (Figure 2D, S13), IAV (Figure 2E, S14), and HCV (Figure S15).

### Non-canonical ORFs discovery

We estimated the discovery of non-canonical ORFs. As part of the pilot library, we included oligos comprising 60 nt upstream and 140 nt downstream of the initiation codons of 716 ORFs identified in HCMV-infected cells by ribosome profiling (20), termed here “Ribo-seq ORFs” (Figure 1B). For each ORF, we also designed a mutated oligo in which we replaced the reported start codon with GCC.

**Figure 3.**
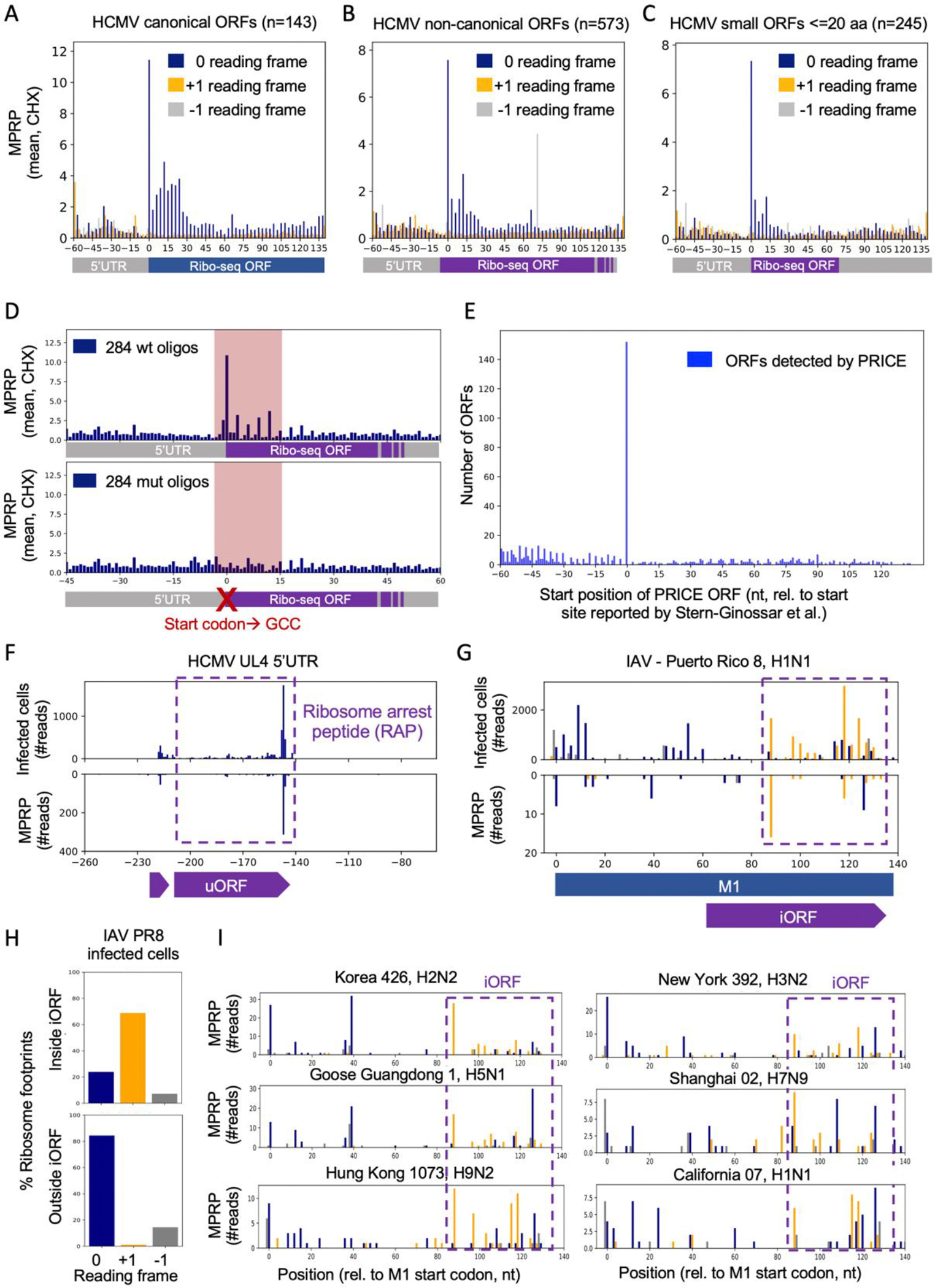
Non-canonical ORFs measurements in MPRP and infected cells **(A-C)** Metagene analysis of oligos containing the sequence of ORFs that were identified by ribosome profiling of HCMV-infected cells (20). Showing the average of ribosome footprints in each position. **(D)** Comparing the average number of ribosome footprints between oligos containing the wt start codon (upper graph) to those in which the reported start codon was mutated to GCC (lower graph). Shown are the average ribosome footprints across 284 oligos, containing Ribo-seq ORFs in the length of 7–45 aa. **(E)** ORF discovery using PRICE. Showing the number of ORFs detected in each position (ORF start position). **(F)** Mirror plot showing the number of ribosome footprints in HCMV-infected cells and MPRP. Purple box highlights uORF2, which encodes a ribosome arrest peptide (21) **(G)** Mirror plot showing the number of ribosome footprints in IAV-infected cells and MPRP. The purple box highlights an internal overlapping iORF in the +1 reading frame. **(H)** Percentages of ribosome footprints mapped to 0, +1, and −1 reading frames in the region encoding the internal ORF (upper panel) and outside this region (lower panel). **(I)** Ribosome footprints on the M1 coding sequences of six additional IAV strains from the MPRP experiment.

We found clear enrichment of ribosome footprints along the reported Ribo-seq ORFs with tri-nucleotide periodicity indicating translation in the correct reading frame. Importantly, MPRP detected ribosome footprints on both canonical and non-canonical Ribo-seq ORFs, including ORFs with a non-AUG start codon and short ORFs in the length of 20 aa or less (Figures 3A–C). Mutating the start codon to GCC of 284 Ribo-seq ORFs resulted in a substantial reduction of ribosome footprints compared to wild-type oligos (Figure 3D). Our findings confirmed the non-AUG start codons reported by Stern-Ginossar et al., (20) and demonstrate how MPRP can be used to functionally characterize the initiation site of non-canonical ORFs. PRICE ORF prediction detected 152 of the 716 Ribo-seq ORFs (21%, accounting for 25% of the total ORFs detected by PRICE) with 11.7-fold enriched initiation at the reported start codon compared to the next abundant position (Figure 3E).

We found similar ribosome footprints on PRICE-predicted uORFs and iORFs in MPRP and cells infected with HCMV, VSV, and IAV (Figures 3F–G, S16). Consistent with HCMV-infected cells, MPRP detected a prominent peak at the end of an uORF in the *UL4* 5’UTR. This uORF was shown to inhibit the translation of the main CDS by causing ribosome stalling (21) (Figure 3F), indicating that MPRP can provide information on functional stalling sites. Ribosome profiling of cells infected with the H1N1 strain of IAV confirms an iORF in the +1 frame of M1 detected by MPRP (Figures 3G–H). Notably, the design of the pan-viral library allowed us to uncover this iORF in six additional influenza strains, including the H5N1 bird flu strain responsible for the current cattle outbreak (Figure 3I).

### Expanding the repertoire of viral antigens

Non-canonical ORFs contribute to the pool of peptides presented on the HLA-I complex to cytotoxic T cells (5, 6, 22–28). Interestingly, peptides from non-canonical ORFs can be enriched on the HLA-I complex in comparison to canonical peptides, with some eliciting stronger T cell responses (7, 26). Thus, non-canonical ORFs discovery can provide new targets for vaccines and insights on the interaction between viruses and the immune system.

**Figure 4.**
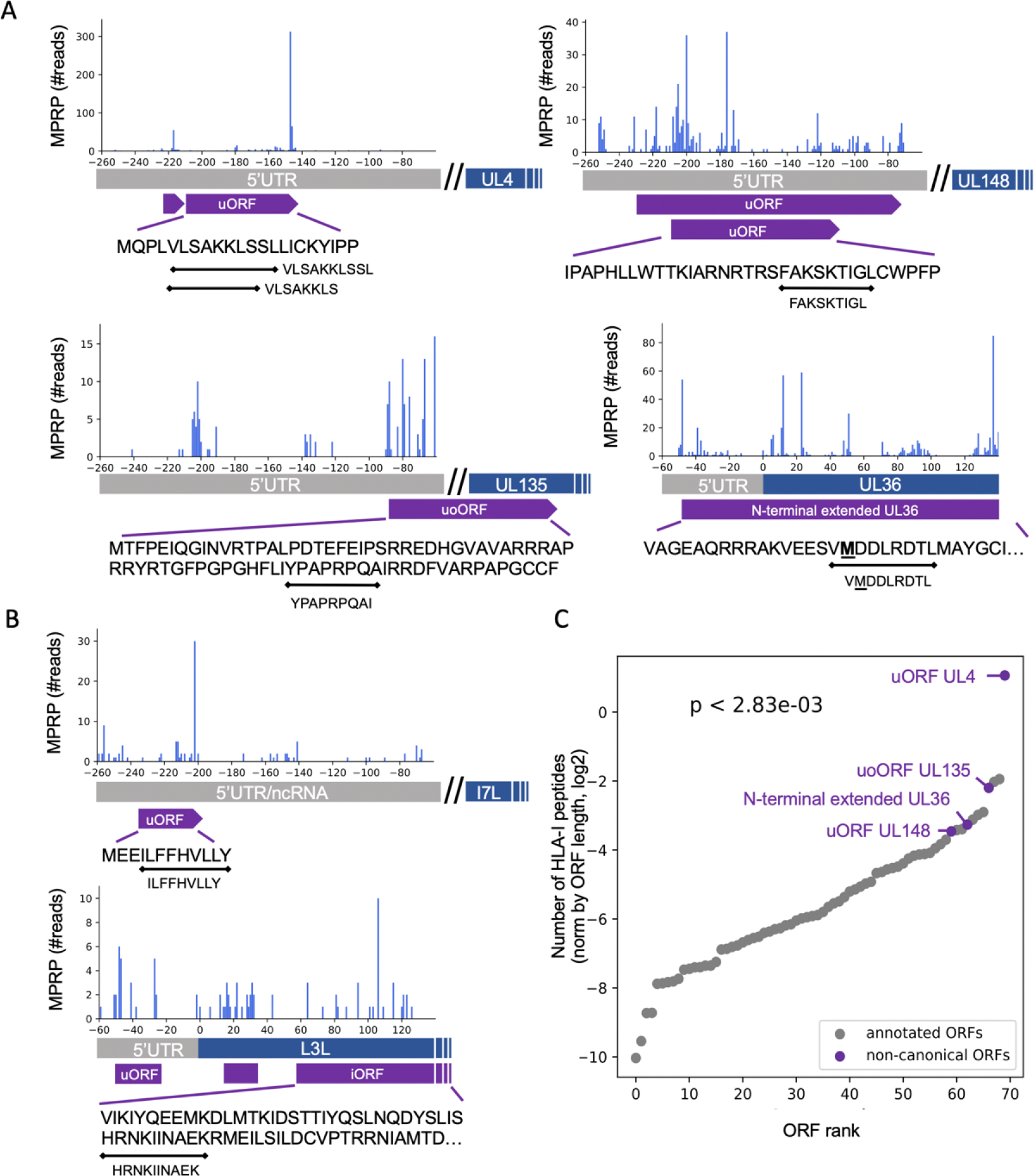
HLA-I peptides derived from non-canonical ORFs in HCMV and VACV **(A)** HLA-I peptides detected in four non-canonical ORFs of HCMV identified by MPRP. **(B)** HLA-I peptides originating from two non-canonical ORFs in VACV: A uORF in the non-coding region upstream of the I7L coding sequence, and an iORF overlapping the coding region of L3L. **(C)** Comparing HLA-I presentation from annotated and non-canonical ORFs in HCMV. For each ORF, we present the number of total HLA-I peptides detected in HCMV immunopeptidome (not unique) normed by the ORF length. p<10^−3^, Wilcoxon rank-sum test.

To assess the contribution of non-canonical ORFs detected by MPRP to HLA-I presentation, we re-analyzed two immunopeptidome datasets from cells infected with either HCMV (19) or vaccinia virus (VACV)(29) (Figure S17). We found five unique peptides from four non-canonical ORFs in HCMV: an uORF in the 5’UTR of UL4 (VLSAKKLS, and VLSAKKLSSL), an uORF in the 5’UTR of UL148 (FAKSKTIGL), an uoORF in the 5’UTR of UL135 (YPAPRPQAI), and an N-terminal extended isoform of the UL36 protein (VMDDLRDTL) (Figure 4A). In VACV-infected cells, we found two unique HLA-I peptides from an ORF located upstream to I7L (ILFFHVLLY) and from an iORF overlapping the coding region of L3L (HRNKIINAEK) (Figure 4B). Six of the seven detected peptides were predicted as good binders by HLAthena (30) (MSi rank <= 2) to at least one of the expressed HLA-I alleles, and all the non-canonical ORFs were supported by a peptide with a good prediction score (Table S4).

Next, we evaluated the contribution of the non-canonical ORFs to HLA-I presentation. Appending MPRP-detected ORFs to the annotated HCMV ORFs increased the number of mapped HLA-I peptides by 7.4% (from 964 to 1035 peptides). Moreover, the non-canonical ORFs produced more HLA-I peptides compared to most of the annotated ORFs (Wilcoxon rank-sum p-value<10^−3^, Figure 4C).

### Exposing uORFs in viral 5’UTRs

**Figure 5.**
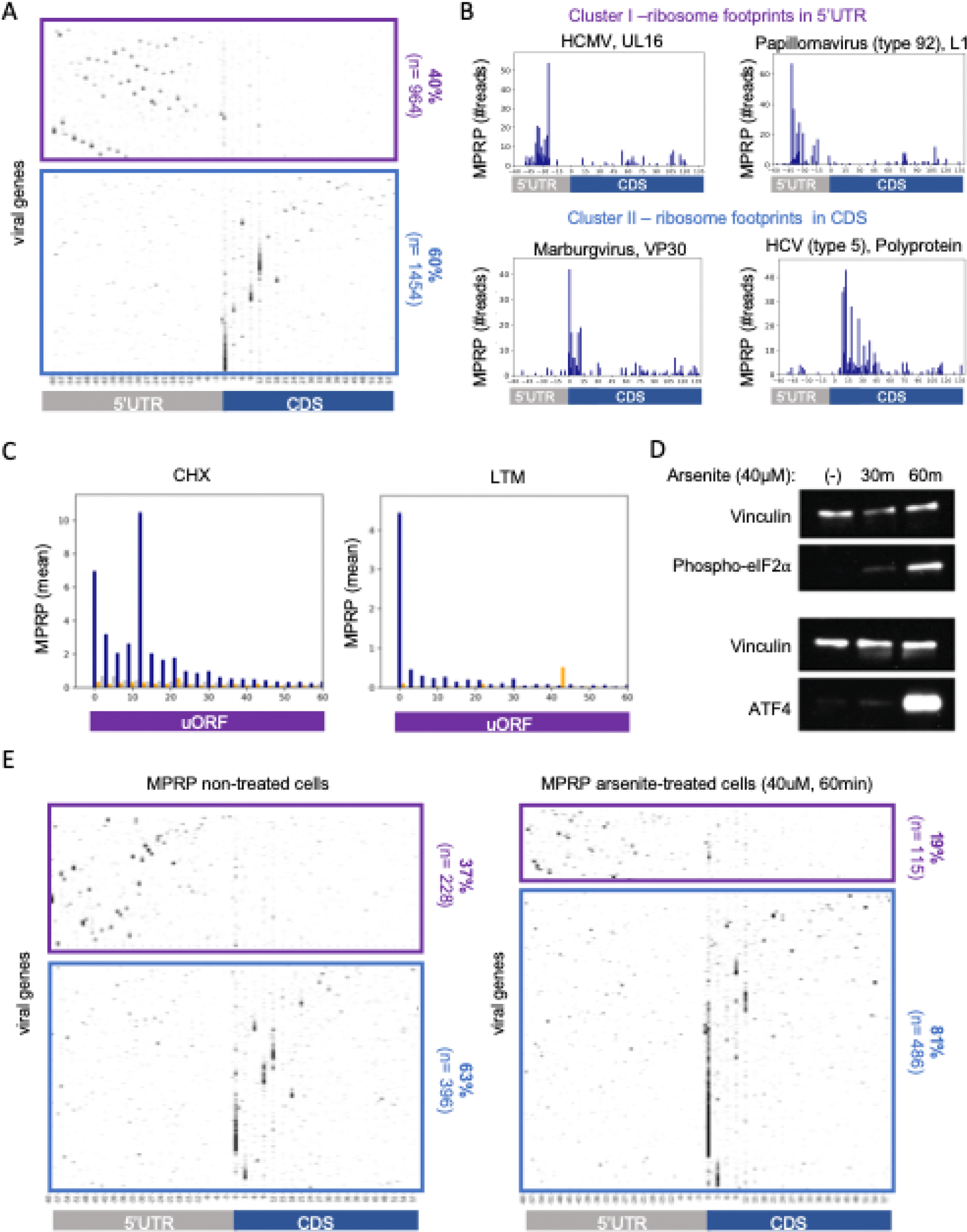
Ribosome densities on uORFs and CDSs in response to eIF2alpha phosphorylation **(A)** Heatmap showing ribosome footprint densities across 2,418 viral oligos. Each line represents a single viral gene and each column represents the position relative to the annotated start codon. Genes in the upper cluster (purple) had more footprints in the 5’UTR region than the CDS region, and genes in the lower cluster (blue) had more footprints in the CDS than the 5’UTR. **(B)** Example of two individual genes from each cluster and the distribution of ribosome footprints observed in each position. **(C)** Metagene analysis showing the average ribosome footprints in each position along uORFs detected by PRICE, relative to the uORF start position. Shown for CHX (left) and LTM (right) inhibitors. **(D)** Western blot analysis of lysates from HEK293T cells treated with 40uM sodium-arsenite for 30 and 60 min. Phosphorylated eIF2alpha was detected with a monoclonal phospho S51 antibody (upper panel). ATF4 protein was detected using a polyclonal antibody (lower panel). In both membranes, Vinculin was used as a loading control. **(E)** Repeating the MPRP experiment in HEK293T cells that were treated with 40uM sodium arsenite and in non-treated cells. Shown are heatmaps of ribosome densities across viral oligos and clusters similarly to the analysis in (A).

uORFs can modulate the expression of viral proteins by attenuating translation initiation at the main CDS (9–11, 31). Examining the distribution of ribosome footprints in 2,418 viral genes, we identified two main clusters: a group of genes in which most of the footprints were observed in the 5’UTR with low occupancy in the CDS region (5’UTR cluster), and a group of genes in which most of the footprints were detected in the CDS with low occupancy in the 5’UTR (CDS cluster) (Figure 5A–B). The tri-nucleotide periodicity observed in uORFs detected in the 5’UTR region indicates that they are actively translated by ribosomes (Figure 5C). Moreover, we found a strong signal of initiating ribosomes at the start codons of these uORFs in the presence of an LTM inhibitor.

We hypothesized that genes in the 5’UTR cluster have uORFs that attenuate translation from the main CDS. uORFs translation response to cellular stress, including viral infection, through the phosphorylation of eIF2alpha (Figure S18). Upon eIF2alpha phosphorylation, pre-initiation complexes (PICs) are more likely to scan through the uORF start codon and initiate translation at the main CDS. To test if the detected uORFs are regulated by cellular stress, we treated cells with sodium arsenite, a potent inducer of eIF2alpha phosphorylation (32). We confirmed increase in eIF2alpha phosphorylation and the uORF-regulated ATF4 protein (Figure 5D).

We performed MPRP in HEK293T cells treated with sodium-arsenite. We found a relative decrease in the fraction of viral genes in which ribosomes were “held” at the 5’UTR (37% to 19% in untreated and treated cells, respectively, Figure 5E) and an increase in the fraction of genes containing ribosome footprints in the CDS (63% to 81% in untreated and treated cells, respectively). This result indicates that in response to eIF2alpha phosphorylation, ribosomes were more likely to bypass the uORFs in the 5’UTR and initiate translation at the main CDS, as expected in the case of inhibitory uORFs.

## DISCUSSION

We present a method to comprehensively screen sequences of many viruses for translated regions in a single pooled experiment. Using MPRP, we uncovered thousands of potential ORFs, and provided high resolution of ribosome footprints across the 5’UTR region and the beginning of the CDS in thousands of viral genes.

While synthetic systems can never fully recapitulate naturally infected cells, MPRP bridges the gap between current computational annotations, which rely on outdated assumptions of ORF characteristics, and traditional ribosome profiling. This is critical given that the landscape of translated regions has not yet been determined for the majority of viruses. MPRP has the capacity to identify translated ORFs in a broad spectrum of viruses and provides a tool for investigating highly pathogenic viruses. Using 200nt-long viral fragments omits the requirement for scarce high-containment facilities. Additionally, we demonstrated how MPRP can provide rapid insights during an outbreak by exposing a non-canonical iORF in the H5N1 bird flu genome. Within a few weeks, MPRP can detect ORFs in a newly discovered virus, independently of its culturing conditions.

We exposed additional sources of viral antigens that could contribute to HLA-I presentation and T cell recognition. While T cell assays almost exclusively assess responses against canonical proteins, some T cell epitopes from non-canonical ORFs induce more potent T cell responses compared with canonical epitopes (7). Thus, the incorporation of non-canonical ORFs into T cell assays has the potential to enhance their sensitivity and facilitate the identification of vaccine targets.

We also discovered viral uORFs that likely affect gene expression regulation. We found numerous potential uORFs that exhibited hallmarks of translation and eIF2alpha phosphorylation responsiveness. These uORFs might function in the temporal regulation of viral proteins as suggested for HHV-6 and HCMV (33). Moreover, MPRP can provide insights into the potential mechanism by which uORFs exert their function, such as ribosome stalling (34).

It is important to acknowledge the limitations inherent in this study. The viral sequences examined here were assessed independently of the broader genome context, and were evaluated in non-infected cells. The 200 nt synthetic oligo might exclude *cis*-regulatory elements such as IRESs (35), VPg proteins (36), uORFs in long 5’UTRs (37), ribosome shunting (38), microRNA binding sites (39), and pseudoknots (40). MPRP does not capture the distinctive biology occurring within cells infected by each of the many viruses studied here and therefore lacks host and/or viral proteins that regulate translation, such as SARS-CoV-2 nsp1(41). Hence, MPRP cannot accurately detect every ORF in each virus.

Nevertheless, we have substantive evidence supporting the identification of genuine ORFs. This evidence includes elongating and initiating ribosomes on annotated and reported non-canonical ORFs, the reproducibility of measurements across different cell types, the notable decrease in ribosome footprints upon mutating AUG and non-AUG start codons, the high similarity of ribosome footprints pattern in MPRP and native virus infection, the corroborative support from HLA-I peptides identified through mass spectrometry, and the responsiveness of uORFs to stress conditions.

In total, our study yields thousands of candidates for unexplored ORFs across hundreds of human viruses, which can enhance our understanding of viral biology and contribute to vaccine development.

## Supplementary Material

Data_Table_S1_pan_viral_library_MPRP_ORFs_PRICE.txt

Data_Table_S2_pan_viral_library_EMCV_IRES_MPRP_ORFs_PRICE.txt

Data_Table_S3_pan_viral_library_Polio_IRES_MPRP_ORFs_PRICE.txt

Data_Table_S4_HLA_peptides_HCMV_and_VACV_non_canonical_ORFs

Supplementary Materials

## Data Availability

The raw sequencing data generated in this study have been submitted to the Gene Expression Omnibus (GEO; https://www.ncbi.nlm.nih.gov/geo/) under accession number GSE272406.
