# Supplementary material for: Pan-viral ORFs discovery using Massively Parallel Ribosome Profiling
Source: Science. Author manuscript; Available in PMC 2025 Sep 24. (PMC12459998; doi:10.1126/science.ado6670)
Supplement: Supplementary Materials [file NIHMS2100651-supplement-Supplementary_Materials.pdf]

Science
AAAS

Supplementary Materials for

Pan-viral ORFs discovery using Massively Parallel Ribosome Profiling

Shira Weingarten-Gabbay, Matthew R. Bauer, Alexandra C. Stanton, Yingpu Yu, Catherine A. Freije, Nicole L. Welch, Chloe K. Boehm, Susan Klaeger, Eva K. Verzani, Daniel López, Lisa E. Hensley, Karl R. Clauser, Steven A. Carr, Jennifer G. Abelin, Charles M. Rice, Pardis C. Sabeti

Correspondence to: shira_weingarten@hms.harvard.edu and pardis@broadinstitute.org

This PDF file includes:

Materials and Methods
Figures S1-S18
References (42-49)
Captions for Data S1 to S4

Other Supplementary Materials for this manuscript include the following:

Data Tables S1 to S4

Materials and Methods

Cell culture

Human embryonic kidney HEK293T cells (female, ATCC CRL-3216) and human lung A549 cells (male, ATCC CCL-185) were maintained at 37ºC and 5% CO2 in Dulbecco's modified Eagle's medium (DMEM) supplemented with 10% fetal bovine serum (FBS) and 1% penicillin and streptomycin.

Library amplification and cloning

ssDNA oligo libraries were synthesized by Agilent Technologies. Each library was dissolved in 100µL Tris 10mM pH 8 and amplified by PCR with specific primers (Fw: GTGAACCGTCAGATCGCCTCGGCACTCCAGTCCT, Rv: AGAGGGTTAGGGATAGGCTTACCTCAGGCTAGTGCGGACCGAGTCG) using NEBNext Ultra II Q5 HotStart (NEB). PCR cycling parameters were set as follows: initial denaturation at 98˚C for 30 seconds, 12 cycles or 98˚C for 10 seconds, 63˚C for 10 seconds, then 72˚C for 10 seconds, followed by a final elongation step at 72˚C for 5 minutes. The library oligos were amplified in 8 50µL reactions, pooled together, and concentrated to 70µL using Amicon Ultra-0.5ml centrifugal filters. The PCR product was then purified using AMPure XP beads (Beckman Coulter) at 1.8X concentration. A pcDNA3.4 backbone was amplified with specific primers (Fw: GGTAAGCCTATCCCTAACCCTCT, Rv: AGGCGATCTGACGGTTCAC) using NEBNext Ultra II Q5 HotStart (NEB). The library insert and linearized backbone were assembled using the NEBuilder HiFi DNA assembly master mix and half of the reaction volume was transformed by electroporation into 100µL of NEB 10-beta electrocompetent E. coli according to the manufacturer's instructions. Transformed cells were allowed to recover for 1 hour at 37˚C in SOC before being grown for 6.5 hours at 37˚C in 100mL LB containing 100µg/mL carbenicillin. A plated dilution series of transformed bacteria was used to verify that the transformation efficiency exceeded 1,000-fold the complexity of the library. Pooled library plasmid was isolated from bacteria using the Qiagen Plasmid Plus Midi Kit.

Library transfection into cells

<u>HEK293T transfection</u>
4 million HEK293T cells were plated in a 10cm tissue culture dish. After 24 hours, cells were transfected with 10µg of library-containing plasmid using X-tremeGENE 9 transfection reagent (Sigma-Aldrich) at a ratio of 3:1 reagent to DNA (30µL transfection reagent for 10µg plasmid) according to the manufacturer's instructions. Ribosome profiling analysis was performed 24 hours post-transfection.

<u>A549 transfection</u>
3 million A549 cells were plated in a 10cm tissue culture dish. After 24 hours, cells were transfected with 15µg of library-containing plasmid using Mirus TransIT-X2 (MIR 6004) transfection reagent at a ratio of 2:1 reagent to DNA (30µL transfection reagent for 15µg plasmid) according to the manufacturer's instructions. Ribosome profiling analysis was performed 24 hours post-transfection.

Ribosome Profiling

Ribosome profiling protocol was adapted from McGlincy and Ingolia(18).

Ribosome footprints purification

10cm dishes of library-transfected HEK293T or A549 cells were treated with 100µg/mL cycloheximide (CHX) for 1 min prior lysis. Cells were washed with ice-cold PBS containing 100µg/mL CHX and lysed on ice by scraping in 400ul lysis buffer (20 mM Tris pH 7.4, 150 mM NaCl, 5 mM $MgCl_2$, 1 mM DTT, 100µg/mL CHX, 1% Triton X-100, 25 U/mL Turbo DNase). Lysates were incubated on ice for 10 min and triturated cells ten times through a 25 gauge needle. Lysates were centrifuged for 10 min at 20000 x g at 4°C and supernatant was removed into a new tube. RNA concentration was determined using a Qubit RNA BR kit. Lysates were aliquoted to cryotubes and flash frozen in liquid nitrogen. 30µg total RNA was digested with 15 units of RNase I (Epicenter) in 200µl polysome buffer (20 mM Tris pH 7.4, 150 mM NaCl, 5 mM $MgCl_2$, 1 mM DTT, 100µg/ml CHX) for 45 min at room temperature. 10uL SUPERase*In were added to stop RNase I digestion and tubes were transferred to ice. Ribosome protected mRNA fragments (RPFs) were enriched using MicroSpin S-400 columns (GE Healthcare, catalog # 27-5140-01). Columns were pre-washed with a 3mL polysome buffer. 100uL of RNAse I digested lysates were loaded to each column and purified RPFs were collected to a new tube by centrifuging the column at 600 x g for 2 min. 400ul TRIzol was added to purified RPFs and RNA was extracted using Direct-zol kit according to manufacturer's instructions, including on column DNase I treatment, and eluted in 50uL water. RNA was participated by adding 38.5ul water, 1.5uL GlycoBlue, 10uL 3M NaOAc pH 5.5 and 150uL isopropanol and incubating on ice for 1hr (or overnight at -20°C). RNA was pelleted by centrifugation for 30 min at 20,000 x g 4˚C, air dried for 10 min and resuspended in 5uL 10mM Tris pH 8. RPFs in the size of 26-34 nt were selected on a 15% polyacrylamide TBE-Urea. RNA was extracted from the gel by adding 400uL RNA gel extraction buffer (300mM NaOAc pH 5.5, 1mM EDTA, and 0.25% SDS), freezing samples for 30 min on dry ice and thawing overnight at room temperature with a gentle mixing nutator. RNA was participated, washed with ice-cold 75% EtOH buffer, air dried and resuspended in 3uL water.

rRNA+WPRE depletion

rRNA was depleted using an RNase H-based digestion protocol that our group developed to enhance the metagenomic detection of RNA virus genomes in clinical and biological samples(42, 43). Our assay detected unexpected ribosome footprints mapped to the WPRE sequence that accounted for a large fraction of the sequencing reads in the MPRP experiment because they originated from a constant region of the overexpressed plasmid. To remove WPRE-derived footprints prior to sample sequencing, we appended probes tailing the WPRE sequence. rRNA-depleted RNA was eluted in 4uL water.

Linker ligation, reverse transcription and cDNA circularization

RNA was dephosphorylated using T4 PNK reaction and ligated to a pre-adenylated linker using T4 RNA ligase. Linkers used for ligation (barcode is highlighted): 5′-/5Phos/NNNNNATCGTAGATCGGAAGAGCACACGTCTGAA/3ddC/ (NI-810), 5′-/5Phos/NNNNNAGCTAAGATCGGAAGAGCACACGTCTGAA/3ddC/ (NI-811), 5′-/5Phos/NNNNNCGTAAAGATCGGAAGAGCACACGTCTGAA/3ddC/ (NI-812), and 5′-/5Phos/NNNNNCTAGAAGATCGGAAGAGCACACGTCTGAA/3ddC/ (NI-813). Unligated linkers were depleted by adding 5'-deadenylase and RecJf to the ligation reaction. Ligation products were purified using the Oligo Clean & Concentrator kit and reverse transcribed using protoscript II and RT primer 5′-/5Phos/NNAGATCGGAAGAGCGTCGTGTAGGGAAAGAG/iSp18/GTGACTGGAGTTCAGACG TGTGCTC (NI-802). cDNA was gel purified using 10% polyacrylamide TBE-Urea gel. cDNA

was extracted from gel by adding 400uL DNA gel extraction buffer (300mM NaCl, 10mM Tris pH 8, and 1 mM EDTA), freezing samples for 30 min on dry ice and thawing overnight at room temperature with a gentle mixing nutator. cDNA was participated, washed with ice-cold 75% EtOH buffer, air dried and resuspended in 12uL 10mM Tris pH 8. cDNA was circularized using CircLigase II.

Library construction PCR and deep sequencing

Sequencing library was PCR amplified from circular DNA using Phusion polymerase and Illumina-adaptor containing primers (10-12 cycles). Primers used: Fw: 5′-AATGATACGGCGACCACCGAGATCTACACTCTTTCCCTACACGACGCTC (NI-798), Rv: 5′-CAAGCAGAAGACGGCATACGAGATCGTGATGTGACTGGAGTTCAGACGTGTG (NI-799). Amplified library was purified from E-gel EX Agarose Gels 4% followed by SPRI beads cleaning. Library was quantified using Qubit dsDNA-HS and analyzed on a TapeStation D1000. Pooled libraries were sequenced on NextSeq 550 System using a NextSeq V2.5 High Output 75 cycle kit (illumina, 20024906).

Ribosome profiling with LTM inhibitor

For LTM treatment, cells were incubated with 10uM LTM for 30 min prior lysis. LTM was also added to PBS and polysome buffer, and lysis buffer at final concentration of 10uM. The rest of the protocol was identical to ribosome profiling with CHX detailed above.

Arsenite, poly(I:C) and thapsigargin treatment

HEK293T cells were treated at 37˚C with 40 uM sodium arsenite (Millipore Sigma, S7400) for 30–60 minutes. HEK293T and A549 cells were treated with 50 nM thapsigargin (Sigma-Aldrich, T9033) for 6 hours, or transfected with 10 ug/mL poly(I:C) (Invivogen, tlrl-pic) using DharmaFECT 1 for 3–12 hours before harvest.

Western blot analysis

HEK293T treated with sodium arsenite were lysed in a 1% SDS lysis buffer. HEK292T/A549 cells treated with poly(I:C) or thapsigargin were lysed in radioimmunoprecipitation assay (RIPA) buffer (Cell Signaling Technologies, 9806S). Both lysis bufferes had protease and phosphatase inhibitors. All protein lysates were centrifuged to remove debris. Total protein concentrations were determined using the Pierce 660 protein assay reagent (Thermo Fisher, 22660) or BCA protein assay kit (Thermo Fisher, 23225). Equal amounts of total protein from each sample were loaded for all western blots.

Proteins were fractionated on 4%–20% Mini-PROTEAN TGX precast polyacrylamide gels (Bio-Rad, 4561096) with tris-glycine-SDS running buffer (25 mM tris, 192 mM glycine, 0.1% SDS, pH 8.3) and transferred to PVDF overnight in a wet transfer tank with Towbin buffer (25 mM tris, 192 mM glycine, 10% v/v methanol). Membranes were blocked for 1 hour in blocking buffer consisting of 5% v/v bovine serum albumin in TBST (TBS + 0.1% Tween-20). All membranes were then incubated for 16 h at 4˚C in one of the following primary antibodies diluted in blocking buffer: rabbit anti-ATF4 (Proteintech 10835-1-AP, 1:1000), mouse anti-EIF2α (Abcam ab5369, 1:1000), rabbit anti-phospho(Ser51)-EIF2α (Abcam ab32157, 1:2000), rabbit anti-PKR (Abcam ab32052, 1:1000), rabbit anti-phospho(Thr451)-PKR (Abcam ab81303, 1:1000), rabbit anti-STAT1 (Proteintech 10144-2-AP, 1:2000), mouse anti-phospho(Tyr701)-STAT1 (BD Biosciences 612233, 1:1000), rabbit anti-IRF3 (Cell Signaling 11904, 1:1000), rabbit anti-phospho(Ser386)-IRF3 (Abcam ab76493, 1:2000), and vinculin (R&D Systems MAB6896, 2 μg/mL). Blots were washed three times for 5 minutes each with TBST and then incubated with a

secondary antibody diluted in blocking buffer for 1 hour at room temperature. Secondary antibodies used were horse anti-mouse HRP (Cell Signaling Technologies 7076P2, 1:5000, for the mouse primary antibodies) and goat anti-rabbit HRP (Abcam ab97051, 1:5000, for the rabbit primary antibodies). Blots were washed three times with TBST, developed with SuperSignal West Pico PLUS substrate (Thermo Fisher, 34580), and imaged on the FluorChem E CCD imager (Protein Simple).

Influenza A virus infection

Our protocol for influenza A viral stock preparation and titering was adapted from(*44*). Influenza (A/Puerto Rico/8/1934(H1N1)) was donated by the laboratory of Daniel Lingwood. To generate a viral stock, the provided virus was diluted 1:1,000 in influenza growth media (DMEM supplemented with 7.5% Albumin Fraction V, 1X PenStrep and TPCK-treated trypsin (final concentration 2 µg/mL)) , and 750 µL of this dilution was added to a T-75 flask of MDCK cells (ATCC, cat# PTA-6500) at 95-100% confluency. Inoculation was performed at 37˚C, 5% CO2 for 1 hour on an orbital shaker. After 1 hour, 12 mL of influenza growth media was added. Virus-containing supernatant was collected when cytopathic effect (CPE) was observed for >75% of the cells (~48-72 hours post infection). The supernatant was then centrifuged to remove cell debris, aliquoted, and stored at -80˚C until use. Viral stocks were titered on MDCK cells by TCID50, and titer was determined by visualization of CPE and the Reed-Muench method.

Similarly, our protocol for influenza infection of A549 cells (ATCC, cat# CCL-185) was adapted from(*44*) . A549 cells were plated on a 10-cm dish and inoculation was performed when the cells reached 75-85% confluency. The cells were infected at an MOI of 5 in 3.5 mLs of titered stock diluted in influenza growth media (described above). Inoculation was performed at 37˚C, 5% CO2 for 1 hour on an orbital shaker. After 1 hour, the cells were washed once with 4 mLs of influenza growth media and then kept in 10 mLs of influenza growth media at 37˚C, 5% CO2 until harvest.

VSV infection

rVSV-GFP was propagated in BsrT7 cells donated by the Sean Whelan lab(*45*, *46*). BsrT7 cells at 85% or 100% confluency were inoculated at an MOI of 3 for 1 h in serum-free media. Cells were rocked on a shaker and after 1 h DMEM-2% FBS was added. Virus-containing cellular supernatant was harvested 24 hpi, centrifuged to remove cell debris, aliquoted, and stored at 80 C until use. rVSV-GFP viral stocks were titered via plaque assay using Vero cells. Vero cells were seeded in a 6-well tissue culture plate 1-2 days prior to inoculation and cultured in cDMEM-10% FBS. Viral stock was diluted via 10-fold dilutions in serum-free cDMEM and inoculation was performed for 1 h, rocking plate either on shaker or manually every 15 min. After 1 h, inoculation media was replaced with methylcellulose overlay media, made as described above. Plaques were counted one day post infection after fixation and staining with a mixture of 0.5% methylene blue and 70% methanol.

HEK293FT cell growth and confluency was assessed by viewing cells at 4x or 10x magnification and used to determine rVSV-GFP viral stock dilution needed for infection. VSV viral stock was diluted in serum-free cDMEM, added to the cells, and the cells were incubated for 1 hr at 37C on a shaker at an MOI of 10. After 1 hr infection, cells were then washed 4 times with cDMEM-2% FBS to remove excess rVSV-GFP and enable measurement of newly produced viral RNA at downstream post-infection timepoints.

HCV infection and ribosome profiling of VSV-infected cells

A highly infectious hepatitis C virus (HCV) genotype 2a clone, J6/JFH Clone2 as described(*47*), was used to generate the HCV stock. Briefly, 10 µg of in vitro-transcribed Clone2 RNA was electroporated into 8×10^6 Huh-7.5.1 cells. The supernatant from the cells, harvested 48-120 hours post-electroporation, was concentrated, and the MOI of the stock was determined by TCID50 using the NS5A antibody 9E10, as described(*48*). An MOI of 1 was used to inoculate 2xP500 Huh-7.5 cells (20 million cells per plate). Seventy-two hours post infection, a ribosome profiling library was prepared following the method described(*49*), with the following modifications: S7 micrococcal nuclease (120 U/ml) was used to produce mRNA-associated monosomes. These monosomes were subsequently isolated using a sucrose density gradient, followed by a sucrose cushion purification step to minimize contamination by other protein-RNA complexes. The ribosome-protect fragments (RPF) were poly(A) adenylated, and the cDNA library was constructed using a poly(T)-containing primer, as described(*12*).

Mapping ribosome footprints to the synthetic library

Illumina sequencing reads were preprocessed prior alignment using FASTX-Toolkit. Illumina adapters were removed using fastx_clipper, reads were split according to samples barcodes using fastx_barcode_splitter.pl and UMI sequence was trimmed using fastx_trimmer. Reads that were mapped to rRNA were removed using bowtie (version 1.2.2). Remaining reads were aligned to an artificial genome composed of all library oligos using bowtie.

ORF discovery using PRICE

We used PRICE to identify ORFs from deep sequencing reads (version 1.0.3)(*19*). PRICE algorithm requires a predefined set of annotated CDSs to estimate the codons that have generated the observed ribosome footprints. When providing the reference genome of the synthetic library, we did not indicate which oligo contains an annotated viral CDS, because we used this information to estimate ORF discovery rate. Instead, we used annotated human CDSs that were translated in library-transfected cells and, thus, were exposed to the exact same experimental conditions (e.g., RNase I concentration and incubation time that can impact footprint size). We generated a chimeric reference genome composed of chromosome 1 in hg19 and an artificial chromosome composed of 15,000 oligos of the pan-viral library. In addition, we generated a gtf file with the annotations of hg19 and library oligos required for PRICE predictions. For each experiment, we mapped deep sequencing reads to the chimeric fasta file using Bowtie. We filtered ORFs with a p-value ≤0.05 after correcting for false discovery rate (FDR). ORFs were then defined by extending each initiating codon to the next in-frame stop codon in the corresponding viral genome.

Tri-nucleotide periodicity analysis

To determine the reading frame, we used ribosome footprints in the exact length of 29nt and plotted the position of the first nt of the sequencing read. Positions were corrected for the P site offset (12nt).

Comparing ribosome footprints on oligos with wild-type and mutated start codon

Since the size of the ribosome footprint is ~29nt, footprints that do not span the region of the three mutated nucleotides are mapped to both the mutated and wt oligo, resulting in similar ribosome occupancy profile downstream of the first few codons. Thus, to estimate the effect of start codon mutations, we computed the number of reads in the region with maximum information, located -3 to +15 relative to the start codon.

Re-analysis of HLA-I immunopeptidome datasets

MS/MS spectra from publicly available immunopeptidomics data were interpreted using Spectrum Mill (SM) v 07.11.216 (proteomics.broadinstitute.org).

Using the SM Data Extractor module for HLA-I immunopeptidomes, spectral merging was disabled, the precursor MH + inclusion range was 600–3000, and the spectral quality filter was a sequence tag length >1 (i.e., minimum of three peaks separated by the in-chain masses of two consecutive amino acids).

Parameters for the SMMS/MS search module for HLA-I immunopeptidomes reported in(*29*), included: no enzyme specificity; precursor and product mass tolerance of ±10 ppm; minimum matched peak intensity of 30%; ESI-ORBITRAP-CID-HLA-v3 scoring; fixed modification: cysteinylation of cysteine; variable modifications: oxidation of methionine, deamidation of asparagine, acetylation of protein N-termini, and pyroglutamic acid at peptide N-terminal glutamine; and precursor mass shift range of −18 to 136 Da. MS/MS spectra were searched against a protein sequence database that contained 310566 entries, including all University of California Santa Cruz Genome Browser genes with hg19 annotation of the genome and its protein-coding transcripts (63,691 entries), 602 common laboratory contaminants, 2043 curated small ORFs (lncRNA and upstream ORFs [uORFs]), 237,427 novel unannotated ORFs (nuORFs) supported by ribosomal profiling nuORF DB v1.037, as well as translated canonical and noncanonical ORFs >7 amino acids observed in the 15K ribosomal profiling library experiment mapping to Cytomegalovirus.

Parameters for the SM MS/MS search module for HLA-I immunopeptidomes reported in Erhard et al.,(*19*) included: no enzyme specificity; precursor and product mass tolerance of ±10 ppm; minimum matched peak intensity of 30%; ESI-QEXACTIVE-HCD-HLA-v3 scoring; variable modifications: cysteinylation of cysteine; oxidation of methionine, deamidation of asparagine, acetylation of protein N-termini, and pyroglutamic acid at peptide N-terminal glutamine; and precursor mass shift range of −18 to 136 Da. MS/MS spectra were searched against a protein sequence database that contained 311427 entries, including all University of California Santa Cruz Genome Browser genes with hg19 annotation of the genome and its protein-coding transcripts (63,691 entries), 602 common laboratory contaminants, 2043 curated small ORFs (lncRNA and upstream ORFs [uORFs]), 237,427 novel unannotated human ORFs (nuORFs) supported by ribosomal profiling nuORF DB v1.037(*25*), as well as translated canonical and noncanonical ORFs >7 amino acids observed in the 15K ribosomal profiling library experiment mapping to Alphavirus Orthopoxvirus.

Using the SM Autovalidation module, peptide-spectrum matches (PSMs) for individual spectra were confidently assigned by applying target-decoy based FDR estimation to achieve <1.0% FDR. For HLA-I immunopeptidomes, PSM-level thresholding was done with a minimum peptide length of 7, minimum backbone cleavage score of 5, and <1.0% FDR across multiple replicates is present. Allowed precursor charges were HLA-I: 1–4. PSMs mapping to non-canonical ORFs were filtered for 8-11mers to perform HLAthena predictions and manually inspected to exclude peptides that were a result of in-source fragmentation (peptides within nested sets with retention times <20 secs apart).

Subset-specific FDR filtering for nuORFs

Subset-specific FDR was performed as previously described(*25*). The aggregate FDR was set to <1% as described above. FDR for the subset of nuORF peptides requires more stringent score thresholding to reach a suitable subset specific FDR of <1%. Subsets of nuORF types were thresholded independently from the HLA dataset through a two-step approach. First, PSM scoring metrics thresholds were tightened on the nuORF subset: minimum SM score of 7, minimum percent scored peak intensity (SPI) of 50%, precursor mass error of ±5 ppm, minimum backbone cleavage score (BCS) of 5. This allows nuORF distributions for each metric to meet or exceed the aggregate distributions. Second, remaining nuORF type subsets with FDR estimate above 1% were further subjected to a grid search to determine the lowest values of BCS and SM score that improved the FDR to <1% for each ORF type in the dataset.

HLAthena HLA-I peptide presentation predictions

HLA peptide prediction was performed using HLAthena(*30*). For HCMV infected HF99-7, HLA A*01:01, A*03:01, B*08:01, B*51:01, C*07:01, C*01:02 were used for HLAthena predictions(*19*). For VACV-infected HOM-2 cells, HLA A*03:01, B*27:05:02, and C*01:02 were used for HLAthena predictions(*29*).

Statistical analyses

To compare the number of ribosome footprints in pairs of oligos representing the same viral sequence with either the wild-type or mutated start codon, we performed Wilcoxon signed rank test. To assess the differences between the number of HLA-I peptides originating from canonical and non-canonical ORFs in HCMV, we performed a non-parametric Wilcoxon rank-sum test. To test if there is a significant overlap between ORFs detected by PRICE when the pan viral library is translated by a cap-dependent or IRES-dependent mechanism, we computed a hypergeometric p-value by counting the number of ORFs detected in each MPRP experiment, the number of ORFs detected in both MPRPs, and the number of total ORFs designed in the library. All correlations reported in the manuscript were computed using Pearson correlation.

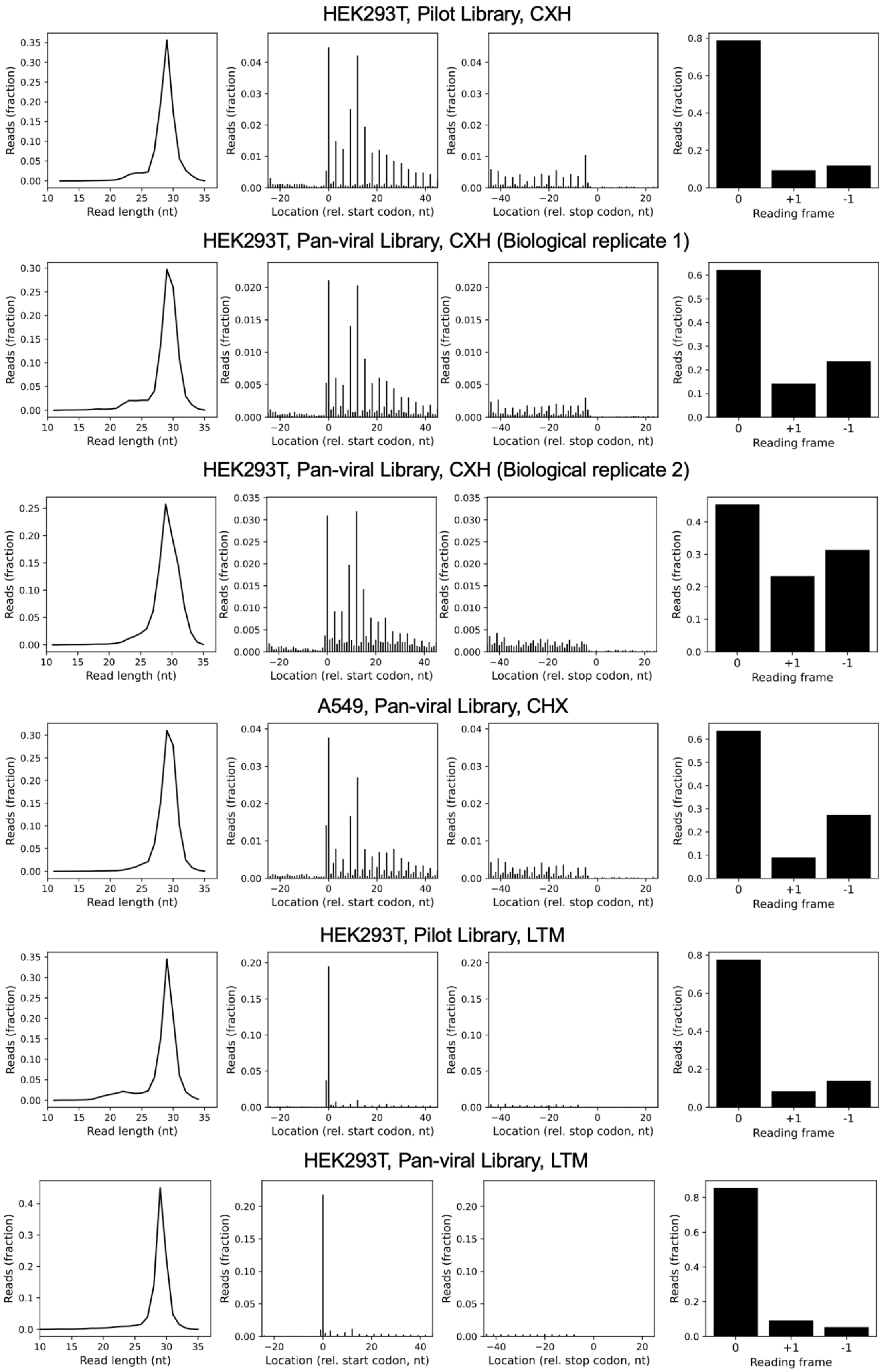
HEK293T, Pilot Library, CXH
HEK293T, Pan-viral Library, CXH (Biological replicate 1)
HEK293T, Pan-viral Library, CXH (Biological replicate 2)
A549, Pan-viral Library, CHX
HEK293T, Pilot Library, LTM
HEK293T, Pan-viral Library, LTM
Reads (fraction)
Read length (nt)
Location (rel. start codon, nt)
Location (rel. stop codon, nt)
Reading frame
0
+1
-1

Figure S1. Quality assurance of ribosome footprints in MPRP experiments

We mapped ribosome footprints to the human genome to estimate the quality of ribosome profiling. Presented for each experiment (left to right): (i) Reads length distribution showing the expected peak at 29-30 nt. (ii) Metagene plots showing lower ribosome occupancies in the 5'UTR relative to the CDS. (iii) Metagene plots showing lower ribosome occupancies in the 3'UTR relative to the CDS. (iv) Fraction of mapped reads to each of the three potential reading frames showing enrichment of the main reading frame.

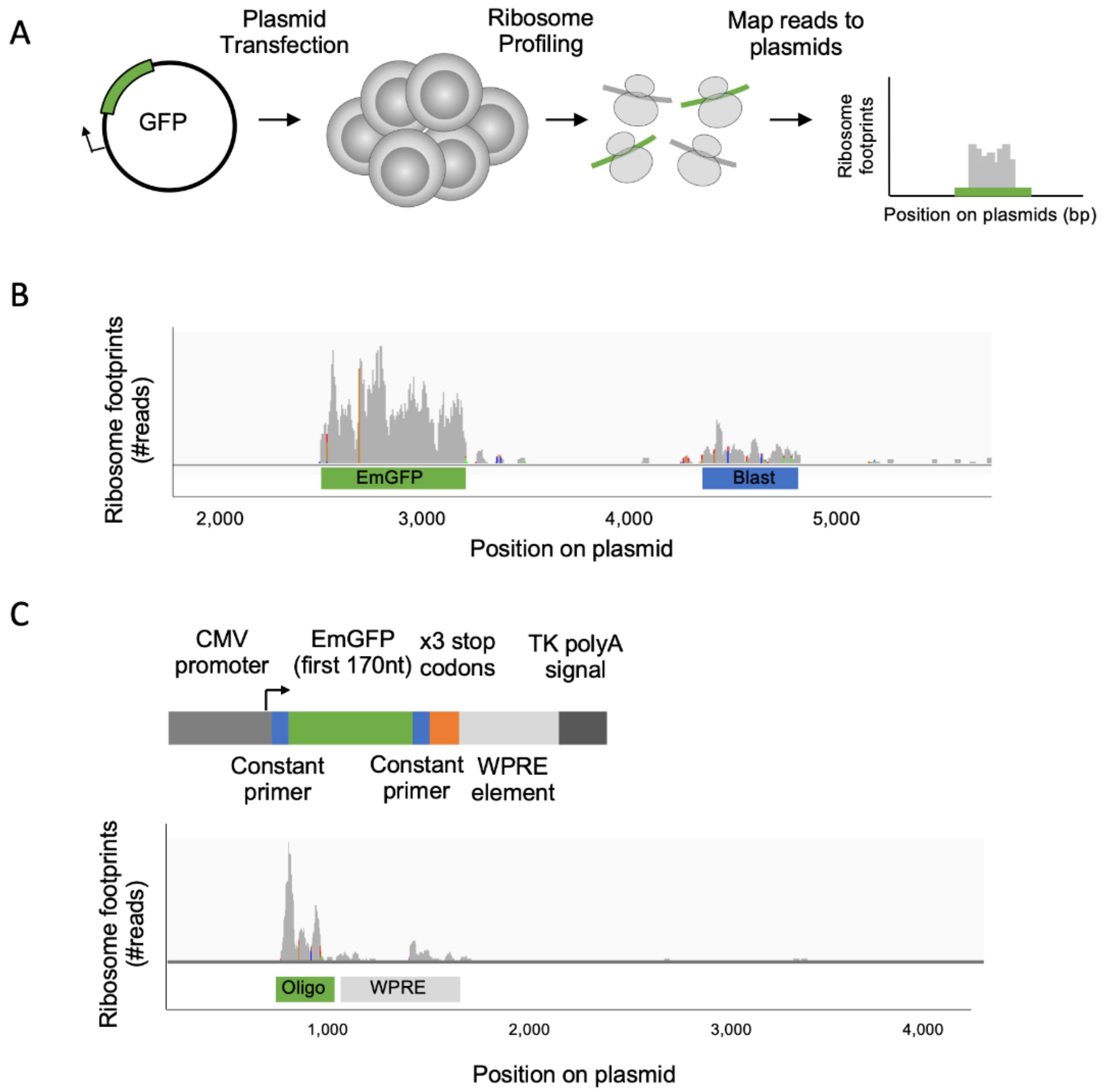

Figure S2. Ribosome profiling of exogenous ORF from overexpression plasmid.
(A) Illustration of EmGFP plasmid transfection followed by ribosome profiling. **(B)** Mapping ribosome footprints to EmGFP plasmids using Integrative Genomics Viewer (IGV). **(C)** (Top) The design of a truncated EmGFP construct mimicking the synthetic library oligos, including the constant primers and the plasmid cloning site. (Bottom) Mapping ribosome footprints to the truncated EmGFP oligo using IGV. Although the cloned sequence contained only part of the original ORF and did not harbor the EmGFP stop codon, we detected ribosome footprints on the truncated GFP regions, with successful termination in one of the three added stop codons.

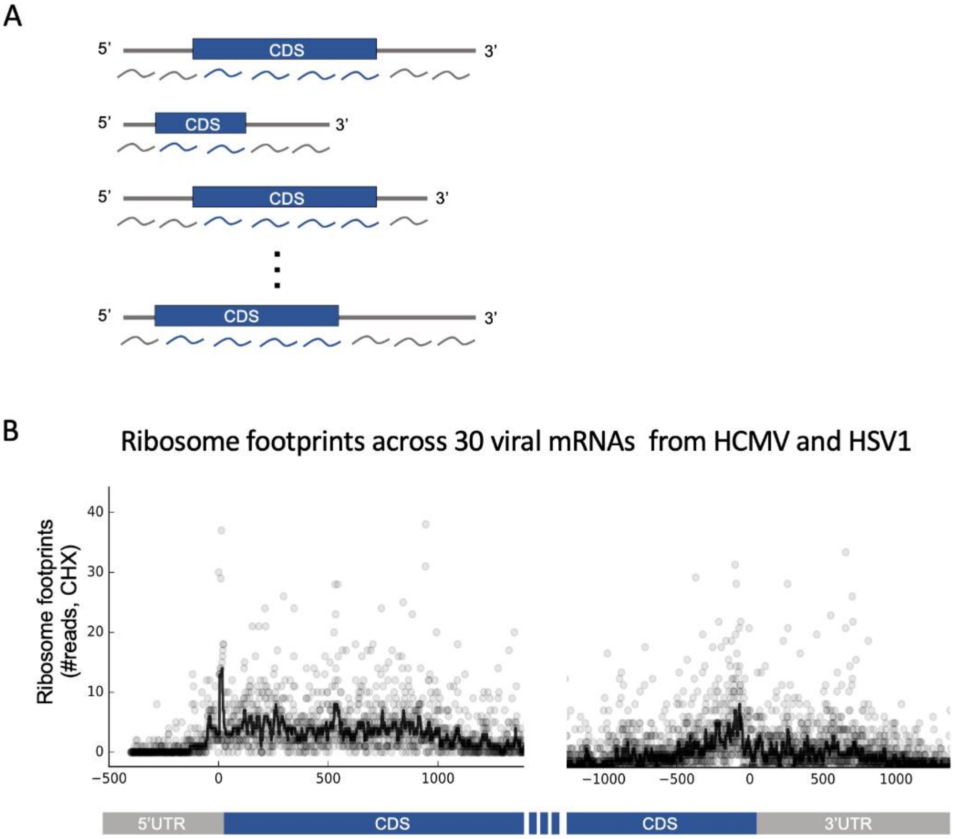

Figure S3. MPRP measurements of tailing oligos across 30 transcripts of HCMV and HSV-1

(A) The design of tailing oligo across 30 mRNAs annotated in the genomes of HCMV and HSV-1 (NC_006273.2 and JN555585.1, respectively). **(B)** Showing the total number of ribosome footprints in each position relative to the CDS start codon or the stop codon in 30 annotated mRNAs (14 of HSV-1 and 16 of HCMV).

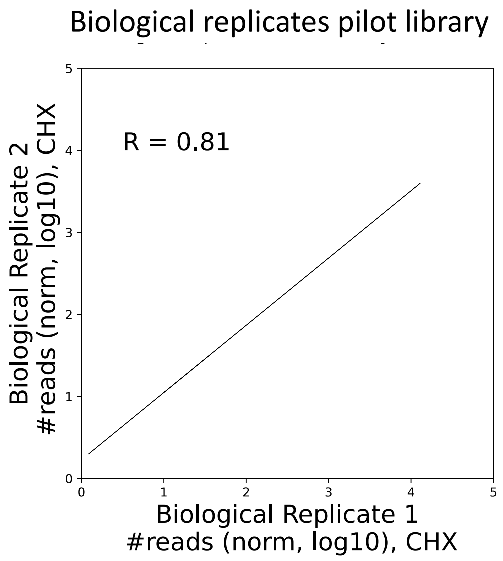

Figure S4. Biological replicate of MPRP measurements of the pilot library

Comparing the number of ribosome footprints mapped to 5,170 oligos of the pilot library in two biological replicates of MPRP experiment in HEK293T cells. R=0.81, Pearson correlation.

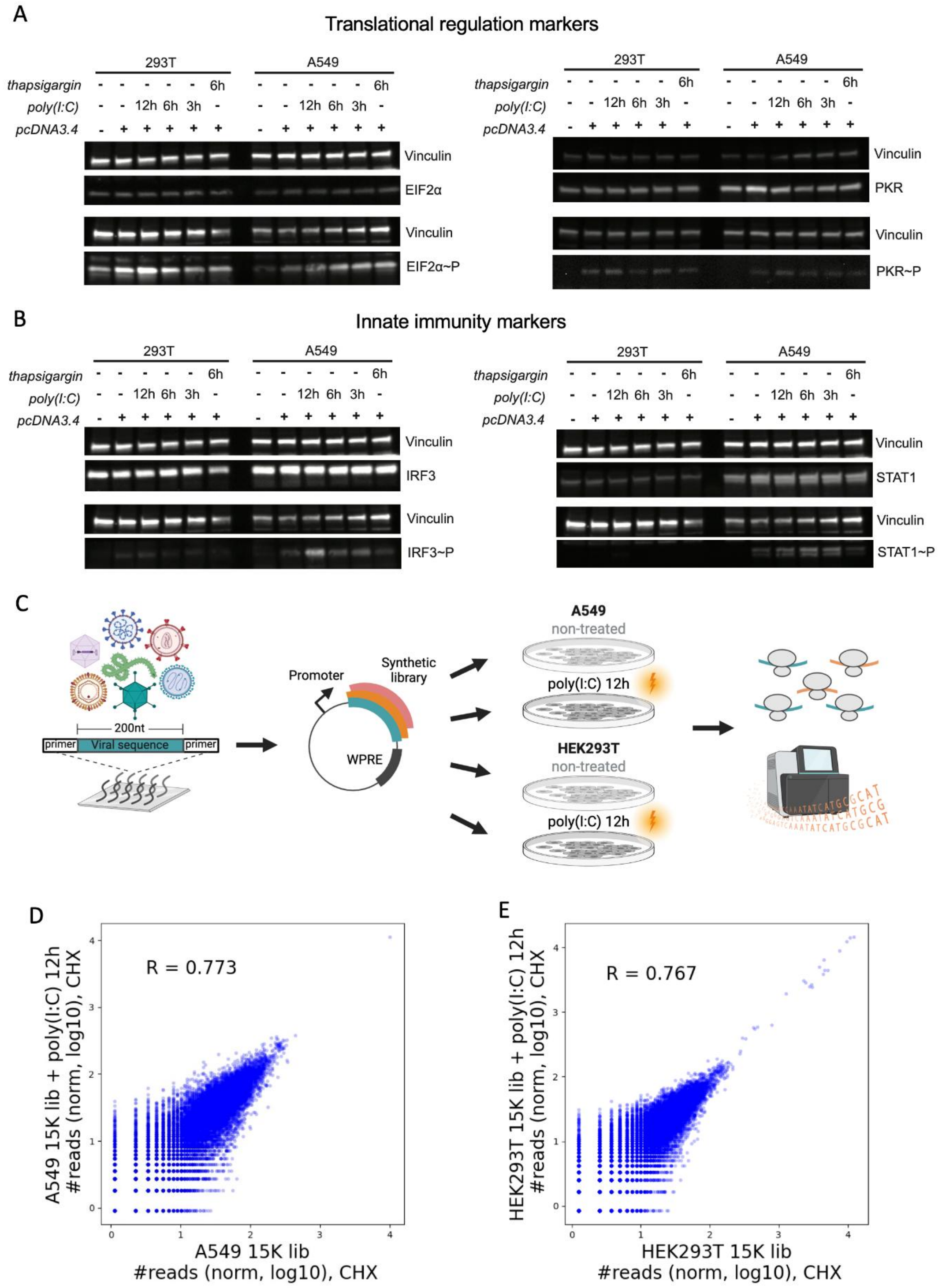

Figure S5. MPRP measurements under stress conditions associated with viral infection
(A-B) Western blot analysis of lysates from HEK293T and A549 cells transfected with pcDNA3.4 plasmid for 24 hours and treated with poly(I:C) or Thapsigargin. For poly(I:C) treatment, 10 ug/mL poly(I:C) were transfected 3, 6, and 12 hours prior to cell lysis. For Thapsigargin treatment, 50 nM Thapsigargin were added 6 hours prior to cell lysis. Showing

protein expression and phosphorylation of translational regulation markers (eIF2alpha and PKR) (A), and innate immunity markers (IRF3 and STAT1) (B). Vinculin was used as a loading control. **(C)** Illustration of MPRP in HEK293T and A549 cells treated with poly(I:C) for 12 hours. **(D)** Comparing the number of ribosome footprints mapped to 15,000 oligos in non-treated and poly(I:C) treated A549 cells. R=0.773, Pearson correlation. **(E)** Comparing the number of ribosome footprints mapped to 15,000 oligos in non-treated and poly(I:C) treated HEK293T cells. R=0.767, Pearson correlation.

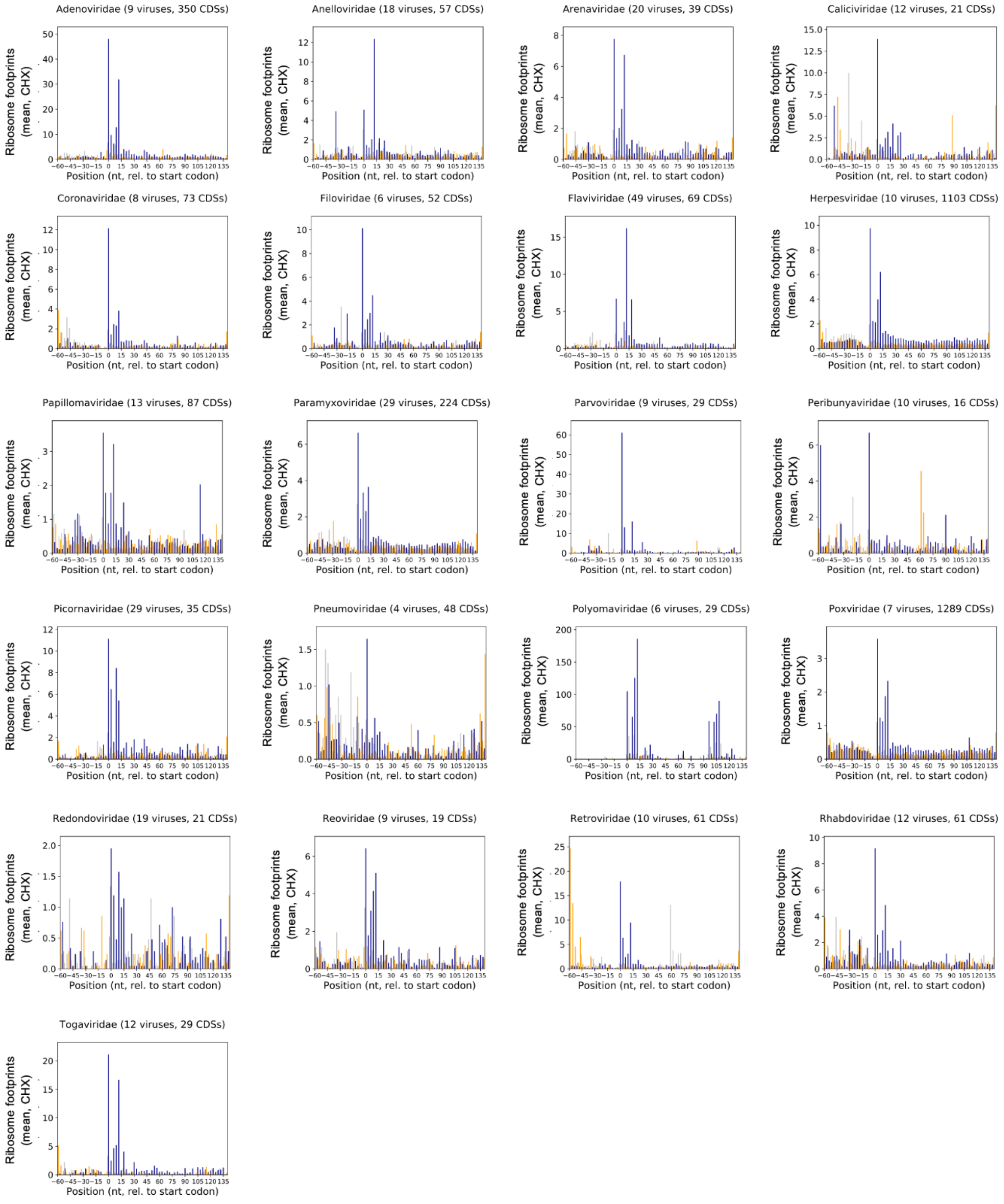

Figure S6. Metagene analysis of elongating ribosome footprints across 21 viral families
The average number of ribosome footprints in each position for 21 viral families observed in MPRP measurements after treatment with CHX to inhibit initiating and elongating ribosomes. Different colors represent the three reading frames (blue, 0; orange, +1; gray, -1)

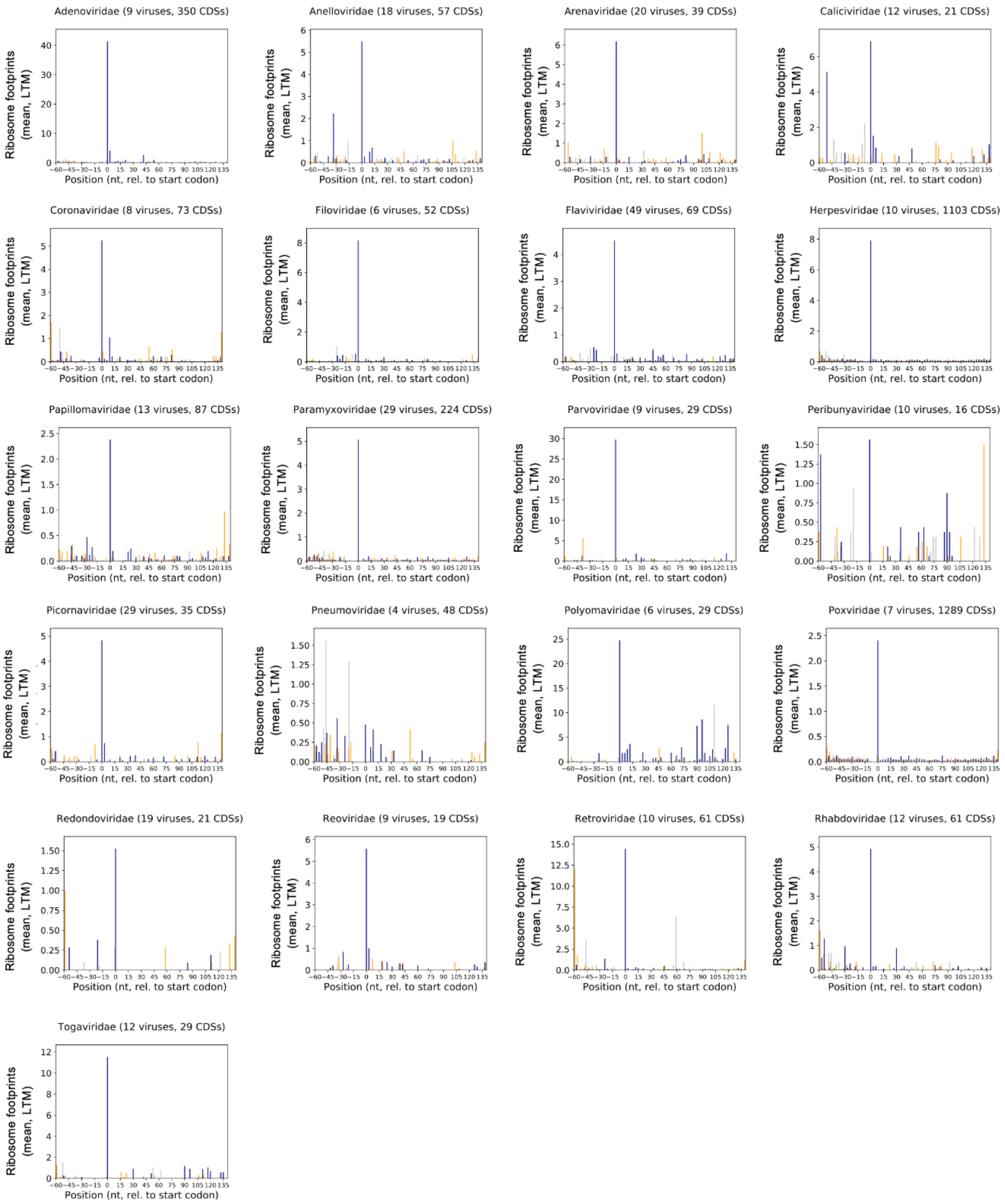

Figure S7. Metagene analysis of initiating ribosome footprints across 21 viral families

The average number of ribosome footprints in each position for 21 viral families observed in MPRP measurements after treatment with LTM to inhibit initiating ribosomes. Different colors represent the three reading frames (blue, 0; orange, +1; gray, -1)

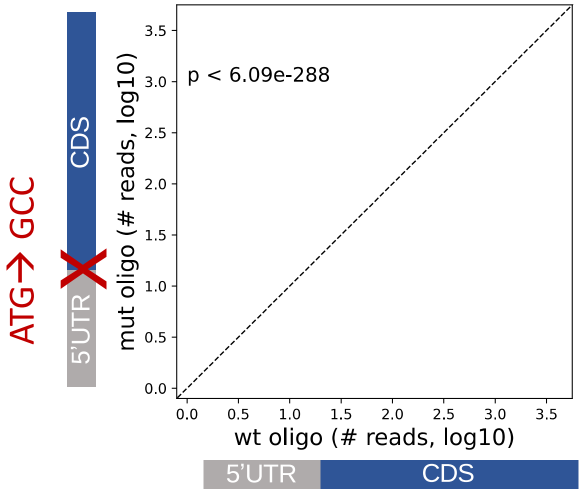

Figure S8. Comparing ribosome footprints on wild-type and mutated start codons
Pairwise analysis of the number of ribosome footprints on 3,777 annotated viral CDSs with either the wild-type start codon or a GCC mutant. Showing the number of footprints in position -3 to +15 relative to the annotated start codon in each oligo. $p<10^{-288}$, Wilcoxon signed-rank test.

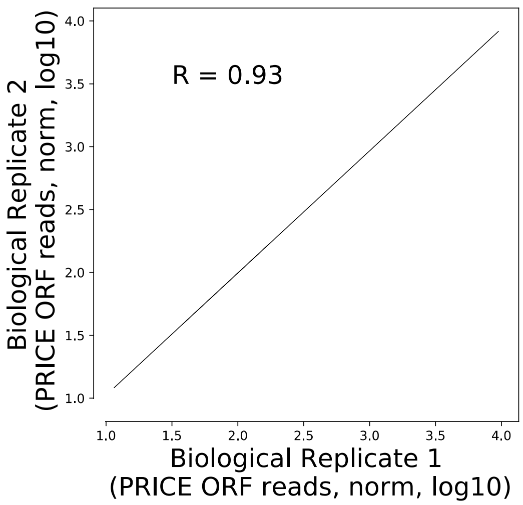

Figure S9. Comparing PRICE translation scores in MPRP biological replicates
The number of reads estimated by PRICE for annotated viral CDSs in two biological replicates of HEK293T cells transfected with the pan-viral library. R=0.93, Pearson correlation.

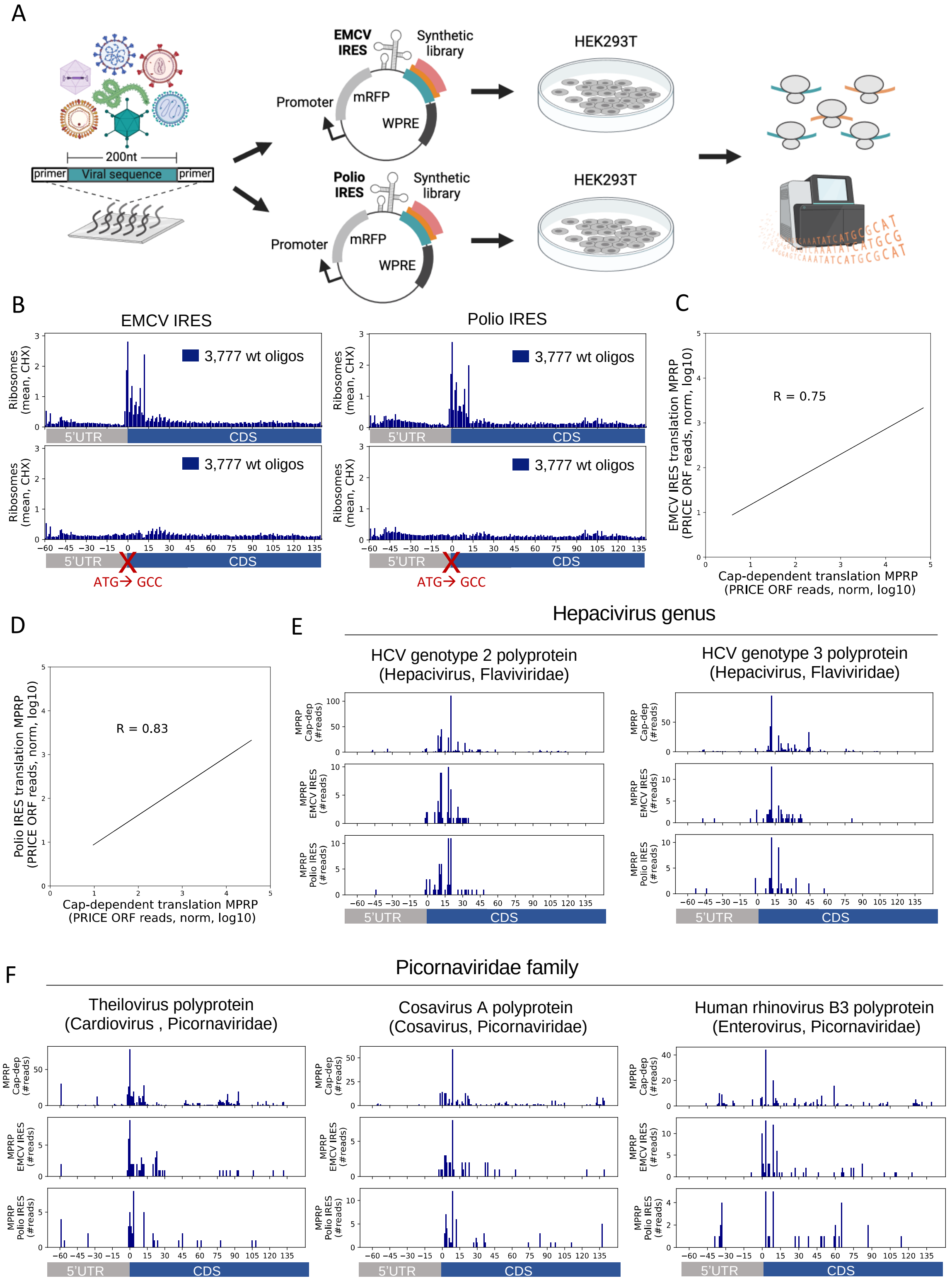

Figure S10. MPRP measurements of the pan-viral library from the EMCV and Polio IRESs **(A)** Illustration of IRES-dependent MPRP. We constructed a bicistronic plasmid with mRFP as the first cistron, driven by cap-dependent translation, and the pan-viral library as the second

cistron, driven by IRES-dependent translation from the EMCV or Polio IRESs. We transfected HEK293T with the two libraries and performed MPRP measurements. **(B)** Comparing the average number of ribosome footprints between oligos containing the wt start codon to those in which the annotated start codon was mutated to GCC. Shown are the average ribosome footprints in each position across 3,777 oligos. The region with maximum information from footprints containing the wild type or mutated start codon (position -3 to +15) is highlighted in red. **(C-D)** Comparing the number of total reads mapped to PRICE ORFs in cap-dependent and IRES-dependent MPRP, for the EMCV IRES (C), and Polio IRES (D). R=0.75 and R=0.83, Pearson correlation. **(E)** Comparing ribosome footprints in the three MPRP experiments (Cap-, EMCV IRES-, and Polio IRES-dependent translation) on oligos containing the first 140nt of polyproteins from Hepatitis C virus genotypes 2 and 3. Both viruses are from the Hepacivirus genus that rely on IRES elements for endogenous translation. **(F)** Similar to (E) for polyproteins from Theilovirus, Cosavirus A, and Human rhinovirus B3 from the Picornaviridae family, also relying on IRES-dependent translation.

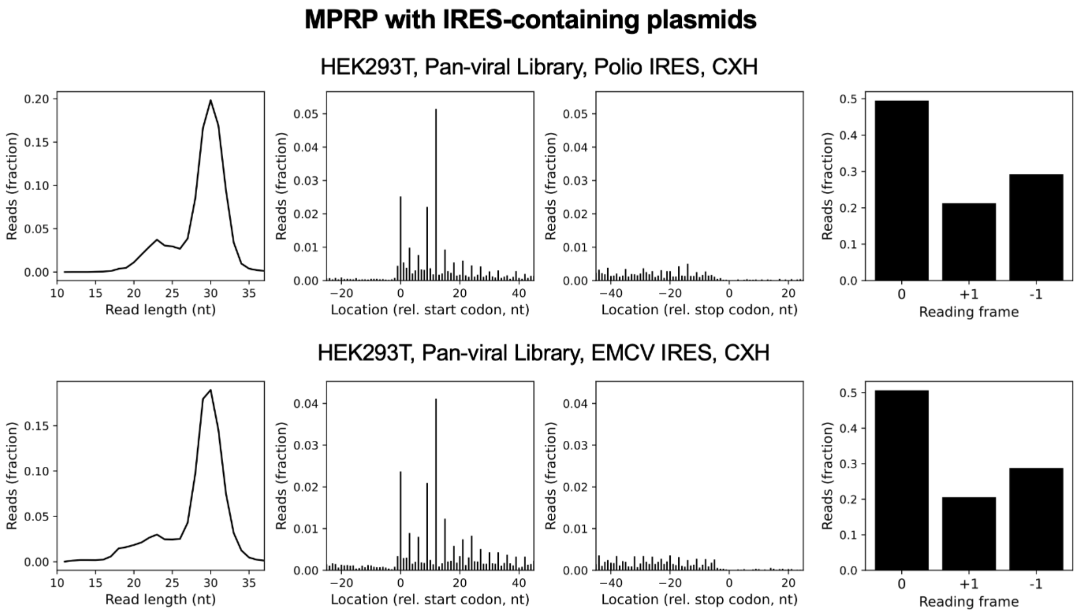

Figure S11. Quality assurance of ribosome footprints in IRES-dependent MPRP
We mapped ribosome footprints to the human genome to estimate the quality of ribosome profiling. Presented for each experiment (left to right): (i) Reads length distribution showing the expected peak at 29-30 nt. (ii) Metagene plots showing lower ribosome occupancies in the 5'UTR relative to the CDS. (iii) Metagene plots showing lower ribosome occupancies in the 3'UTR relative to the CDS. (iv) Fraction of mapped reads to each of the three potential reading frames showing enrichment of the main reading frame.

A

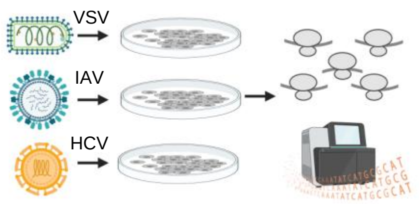

B

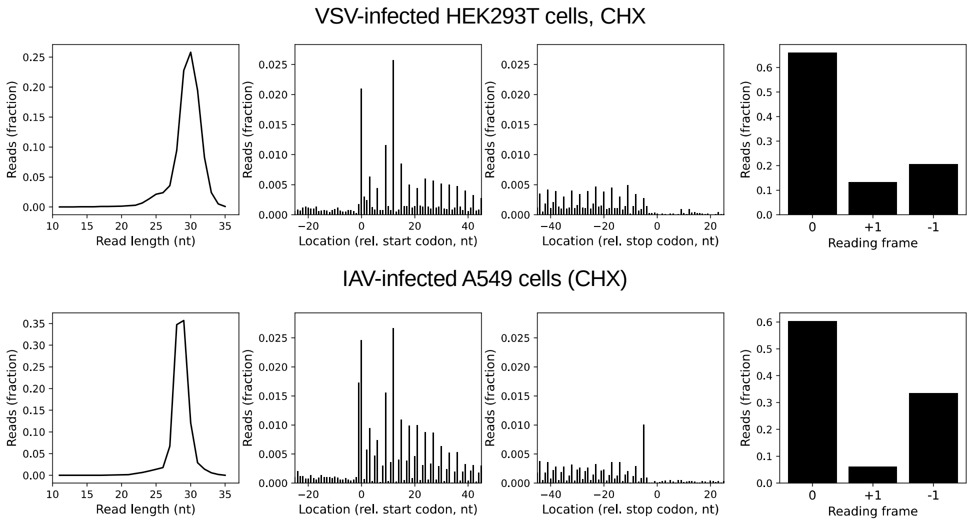

Figure S12. Ribosome profiling of infected cells

(A) We performed ribosome profiling of HEK293T cell infected with VSV (MOI=10), A549 cells infected with IAV (MOI=5), and Huh-7.5 cells infected with HCV (MOI=1). **(B)** Quality assurance of ribosome footprints in VSV- and IAV-infected cells. We mapped ribosome footprints to the human genome to estimate the quality of ribosome profiling. Presented for each experiment (left to right): (i) Reads length distribution showing the expected peak at 29-30 nt. (ii) Metagene plots showing lower ribosome occupancies in the 5'UTR relative to the CDS. (iii) Metagene plots showing lower ribosome occupancies in the 3'UTR relative to the CDS. (iv) Fraction of mapped reads to each of the three potential reading frames showing enrichment of the main reading frame. HCV-infected cells were profiled using MNase, which has a bias toward the 5' of A nucleotides and doesn't provide subcodon resolution. For this reason, we did not perform tri-nucleotides analysis on this sample.

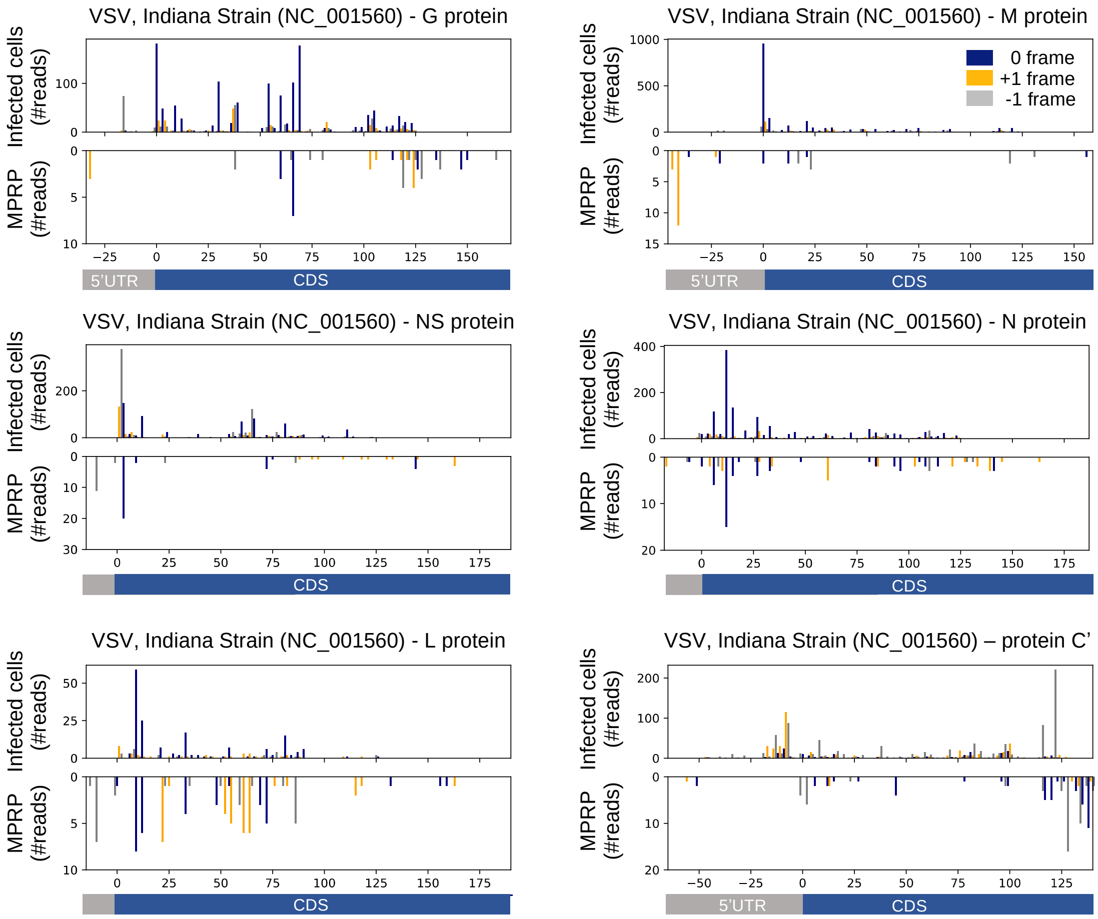

Figure S13. Comparing MPRP to VSV-infected cells

Mirror plots comparing the number of ribosome footprints obtained from ribosome profiling of cells infected with VSV (upper panel) and MPRP (lower panel) for oligos representing the beginning of all VSV coding sequences and the 5'UTRs.

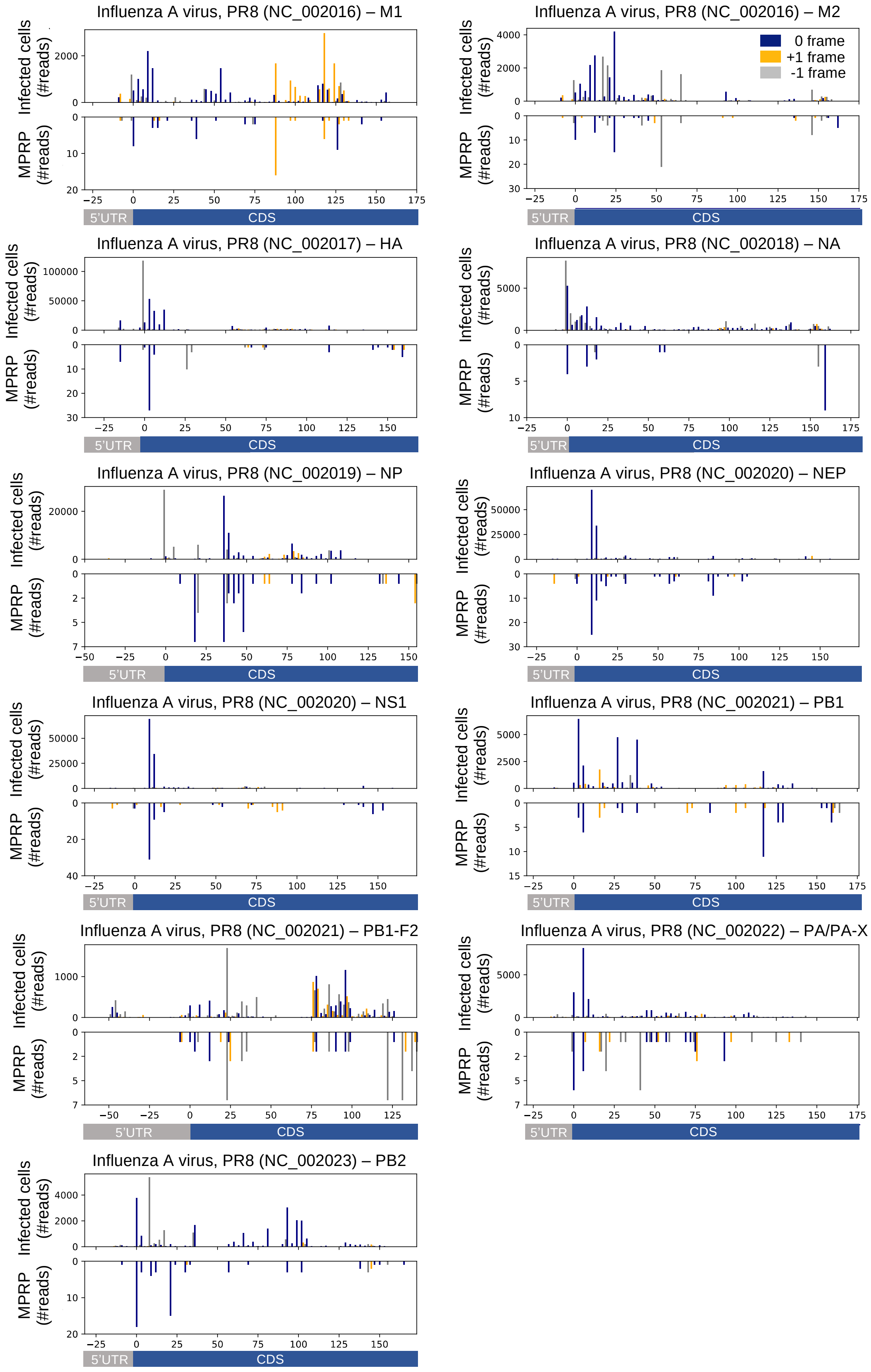
Influenza A virus, PR8 (NC_002016) – M1
Influenza A virus, PR8 (NC_002016) – M2
0 frame
+1 frame
-1 frame
Influenza A virus, PR8 (NC_002017) – HA
Influenza A virus, PR8 (NC_002018) – NA
Influenza A virus, PR8 (NC_002019) – NP
Influenza A virus, PR8 (NC_002020) – NEP
Influenza A virus, PR8 (NC_002020) – NS1
Influenza A virus, PR8 (NC_002021) – PB1
Influenza A virus, PR8 (NC_002021) – PB1-F2
Influenza A virus, PR8 (NC_002022) – PA/PA-X
Influenza A virus, PR8 (NC_002023) – PB2
Infected cells (#reads)
MPRP (#reads)
5'UTR
CDS

Figure S14. Comparing MPRP to IAV-infected cells

Mirror plots comparing the number of ribosome footprints obtained from ribosome profiling of cells infected with IAV (upper panel) and MPRP (lower panel) for oligos representing the beginning of all IAV coding sequences and the 5'UTRs.

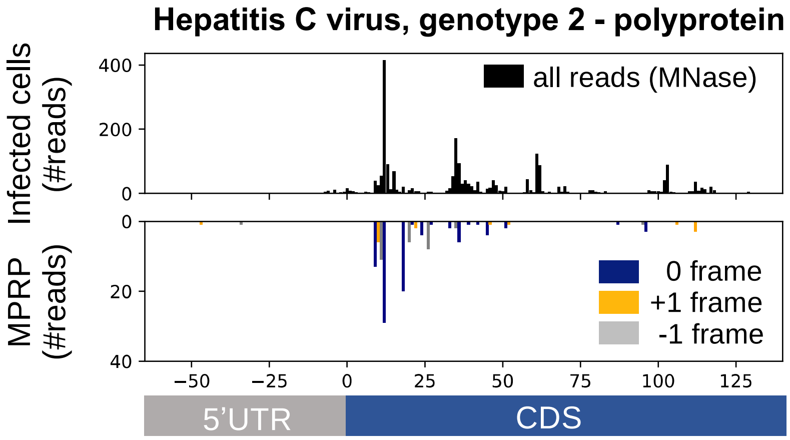

Figure S15. Comparing MPRP to HCV-infected cells

Ribosome profiling was performed on Huh7.5 cells infected with HCV genotype 2 (MOI=1). Showing a mirror plot comparing the number of ribosome footprints mapped to the last 60nt of the HCV 5'UTR and the first 140nt of polyprotein coding sequence. In contrast to MPRP, in which we used RNase I to generate ribosome footprints, HCV-infected cells were profiled using MNase, which has a bias toward the 5' of A nucleotides and doesn't provide subcodon resolution. For this reason, we plotted all mapped reads and did not infer the reading frame as we did for MPRP.

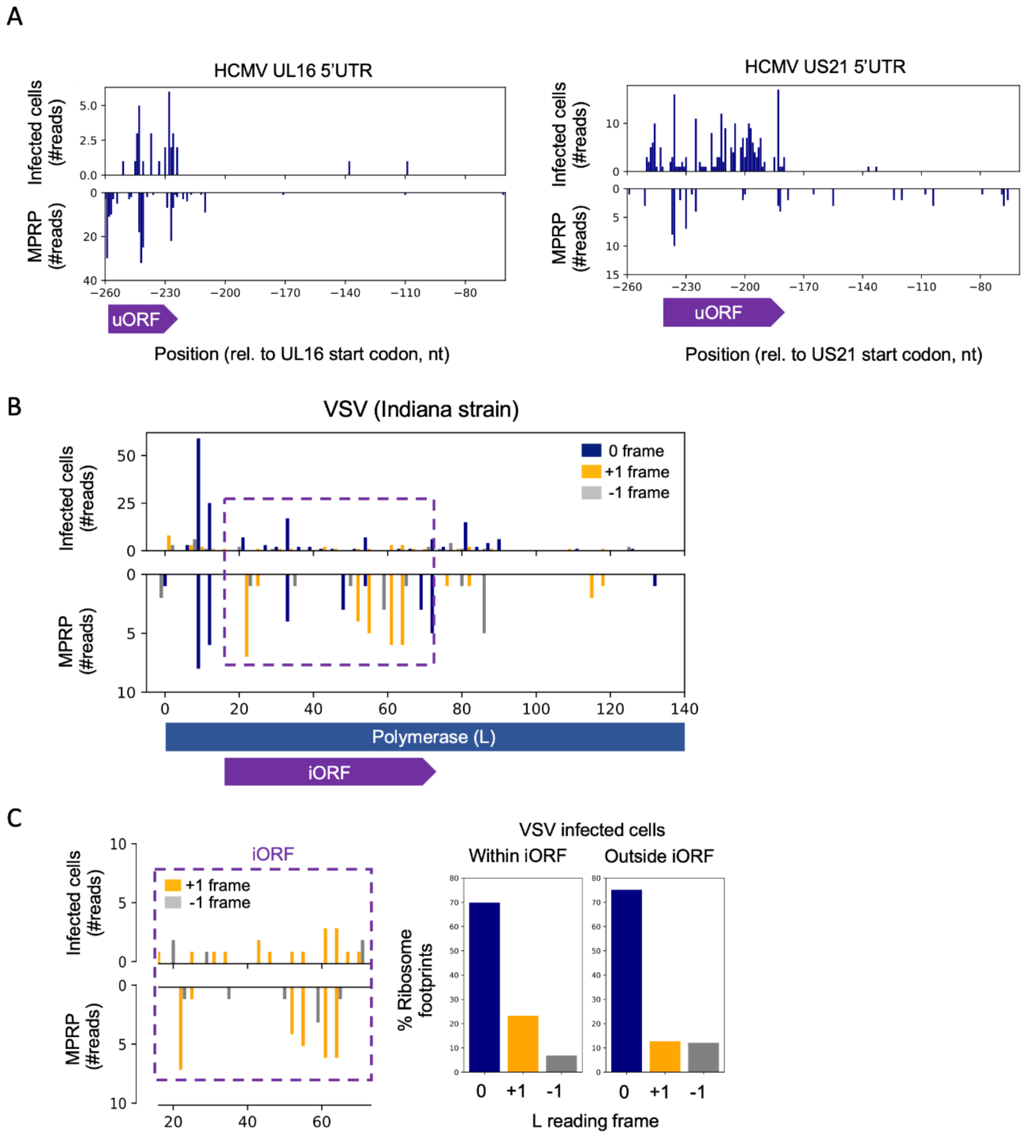

Figure S16. Comparison of ribosome footprints mapped to non-canonical ORFs in MPRP and infected cells

(A) Mirror plot showing the number of ribosome footprints mapped to sequences from the 5'UTRs of *UL16* and *US21* in HCMV-infected cells and MPRP. **(B)** Mirror plot showing the number of ribosome footprints mapped to the first 140nt of the polymerase coding sequence in VSV-infected cells and MPRP. The purple box highlights an internal overlapping ORF detected by MPRP in the +1 reading frame. **(C)** (left) Zoomed-in view of the iORF shown in (B). To facilitate visual comparison of footprints in the alternative reading frames, footprints originating from the main reading frame were removed. (right) Percentages of ribosome footprints mapped to 0, +1, and -1 reading frames in the region encoding the internal ORF and outside this region.

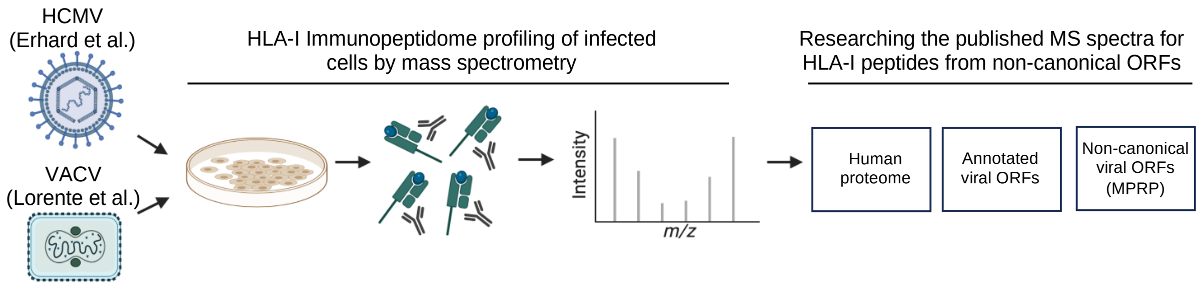

Figure S17. Analysis of HCMV and VACV HLA-I immunopeptidomes

Illustration of the HLA-I immunopeptidome profiling done by Erhard et al., and Lorente et al. We appended non-canonical ORFs detected by MPRP to the search dataset and re-searched the raw data files generated by these two studies.

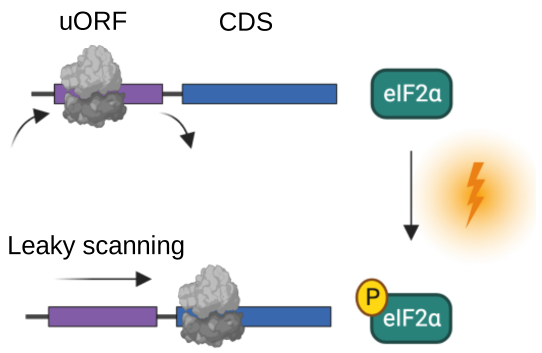

Figure S18. Illustration of uORF-mediated translation regulation in response to stress
In non-stressed cells, the pre-initiation complex (PIC) initiate translation at the uORF start codon, reducing translation of the main CDS. In response to cellular stress, eIF2alpha is phosphorylated. PIC scans through the uORF start codon and initiate translation at the CDS start codon.

Data Table S1. (separate file)

ORFs detected in MPRP measurements of the pan-viral library expressed by cap-dependent translation

Data Table S2. (separate file)

ORFs detected in MPRP measurements of the pan-viral library expressed by the EMCV-IRES

Data Table S3. (separate file)

ORFs detected in MPRP measurements of the pan-viral library expressed by the Polio-IRES

Data Table S4. (separate file)

HLA-I peptides originating from non-canonical ORFs in HCMV and VACV
